# Learning molecular traits of human pain disease via voltage-gated sodium channel structure renormalization

**DOI:** 10.1016/j.csbj.2025.11.048

**Published:** 2025-12-01

**Authors:** Markos N. Xenakis, Angelika Lampert

**Affiliations:** a Institute of Neurophysiology, Uniklinik RWTH Aachen, Pauwelsstraße 30, Aachen, 52074, NRW, Germany; b Scientific Center for Neuropathic Pain Research Aachen, SCNAACHEN, Uniklinik RWTH Aachen, Roermonderstraße 110a, Aachen, 52072, NRW, Germany

**Keywords:** Voltage-gated sodium channel, Renormalization, Criticality, Human pain disease, Machine learning

## Abstract

Mammalian neurophysiology vitally depends on the stable functioning of transmembrane, pore-forming voltage-sensing proteins known as voltage-gated sodium channels (NaVChs). Deciphering the principles of NaVCh spatial organization can illuminate fundamental structure-function aspects of pore-forming proteins and offer new opportunities for pharmacological treatment of associated diseases such as chronic pain. Here, we introduce a renormalization group flow paradigm permitting a formal investigation of NaVCh thermostability properties. Our procedures are solidified by deriving an atom-packing entropy and validated over 121 experimentally resolved NaVCh structures of prokaryotic and eukaryotic origin. We uncover the universality of a critical inflection point regulating the thermostability of the pore domain relative to the voltage sensors, summarized in terms of a generalized Widom scaling law. A machine learning algorithm, rationalized in terms of the violation of inertia and conductivity channel constraints, identifies pain-disease-associated mutation hotspots in the human NaV1.7 channel. Our work illustrates how first-principles-based machine learning approaches can deliver accurate insights for human pain medicine and clinicians at a reduced computational cost, while clarifying the self-organized critical nature of NaVChs.

## Graphical Abstract

**Figure:**
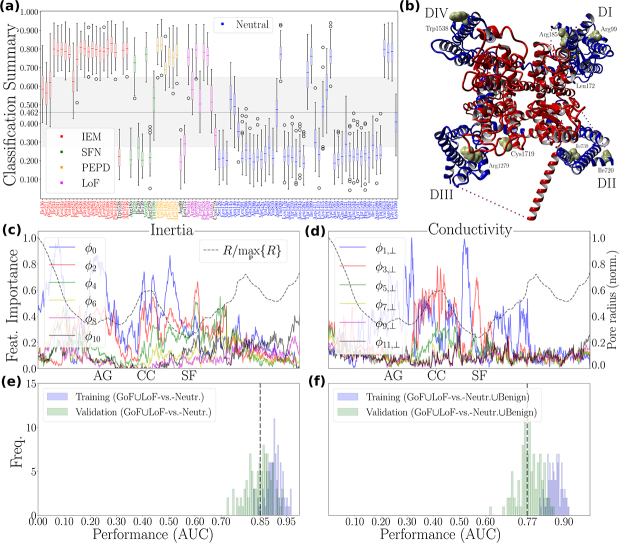

## 1 Introduction

Voltage-gated sodium channels (NaVChs) play a central role in neurophysiology by initiating and propagating action potentials along neuronal tissue [1]. Functionally, they act as ‘traffic controllers’ for sodium ions crossing the cell membrane, thereby shaping the upstroke of the action potential [2], [3].

A key feature that makes sodium transport entropically favorable within the hydrophobic membrane is hydrophilicity [4]. Hydrophilic groups coordinate the dehydration of sodium ions as they traverse the NaVCh pore, enabling efficient permeation [5], [6], [7]. The prevailing view is that NaVCh selectivity arises from a finely tuned balance of strong and potentially long-range interactions between the selectivity filter (SF) and surrounding residue clusters [8], a concept supported by early electrophysiological studies [9].

Members of the NaVCh superfamily share a conserved structural organization: four radially arranged homologous domains (DI–IV) form a porous membrane environment [10]. The first NaVCh structure solved was the prokaryotic channel from *Arcobacter butzleri* (NaVAb), captured in a pre-open state [11]. As predicted from previous crystallographic insights into potassium channels [12], [13], NaVAb confirmed that each domain contains a pore module (PM) linked to a voltage-sensor domain (VSD) [11]. The PM consists of two antiparallel α-helical segments (S5–S6), connected by an extracellular loop and the SF [11]. Together, the four PMs form the pore domain (PD), composed of a narrow, sieve-like SF region, a hydrophobic central cavity, and an intracellular constriction where the putative activation gate (AG) resides [11].

The VSD comprises four transmembrane α-helices (S1–S4) and is connected to the PM through an S4–S5 linker [11]. Positively charged residues in S4 detect membrane depolarization [14]; their outward displacement exerts a pulling force that opens the pore [14], [15], [16], [17]. Compared with their prokaryotic ancestors, eukaryotic NaVChs display greater structural and functional diversity, reflecting the breaking of radial symmetry [10]. As a result, eukaryotic channels exhibit a richer, more specialized metastable dynamics repertoire and enhanced allosteric efficiency [18], [19], [20].

Complex biomolecules, such as NaVChs, have evolved to balance sensitivity and robustness to perturbations of environmental and genetic origin [21], [22]. For example, the gnomAD database [23] reports thousands of benign variants in human NaVCh genes, such as 729 benign variants in *SCN9A* [24], which encodes the pain-related NaV1.7 channel [25]. This abundance of tolerated variation indicates that NaVChs possess substantial structural and functional resilience. What physical principle enables this?

Protein systems often rely on self-similarity, or scale invariance [26], to optimally distribute internal energy and external stresses across their structure [27], [28]. This property is characteristic of self-organized criticality (SOC) [29], an evolutionary strategy that preserves high functional sensitivity while maintaining robustness against mutations [21], [22], [27], [28], [30], [31].

Building on early work showing universal transient behavior in the hydropathic radial profile of globular proteins [53], [54], [55], [56], it was suggested that SOC signatures in proteins manifest as patterns of extrema, peaks and valleys, in intrinsic thermostability cost functions linked to water-mediated interactions [30], [31]. Scaling analysis of the atomic environment around the NaVAb and NaV1.7 pores demonstrates that the atomic distribution around the pore is predominantly unimodal, i.e., that the corresponding cumulative distribution function admits a prominent inflection point [34], [35]. In addition, at specific pore points (such as those marking the SF pore region) the hydropathic dipole field magnitude increases self-similarly along the radial direction, exhibiting a distinct peak near this inflection point [34], [35]. Also, the radial location of the inflection point corresponds to the characteristic size of the PD, marking the structural transition from the PD to the voltage-sensor domains (VSDs) [34], [35].

This threefold ‘coincidence’ – the unimodal atomic distribution, the self-similar increment of the hydropathic dipole field magnitude, and the existence of a characteristic molecular length scale describing the transition from the PD to the VSDs – suggests that such inflection points may serve as subtle indicators of SOC [32]. It also supports a conceptual analogy between the spatial atom arrangement surrounding the pore and the archetypal SOC sandpile model [29], offering a useful toy model for conceptualizing NaVCh molecular complexity [33]. Accordingly, residue structural positions are abstracted as lattice sites, with mutations analogous to externally added sand grains perturbing the lattice structure. The slope of the sandpile, inferred from scaling (power-law) exponents [30], [31], [34], [35], predicts whether a mutation at a given site is likely to destabilize the molecule, thereby providing a rationale for distinguishing between pain-disease-associated and benign structural locations in NaV1.7 [35].

A self-consistent scaling theory for proteins must rest on a renormalization group (RG) foundation [36]. Here, we examine this assumption within the NaVCh superfamily and show how it can yield biomedically relevant insights into inherited human pain disorders. Starting from the simplest radial differential equation that can rationally support a pore-forming architecture with a PD/VSD interface, we derive an RG flow equation that enables a substantial yet biophysically meaningful reduction of atom-packing degrees of freedom (DoF) around a NaVCh pore. ‘Atom-packing DoF’ refers to the number of positional possibilities available to atoms surrounding a pore point at any moment. Beyond traditional definitions of molecular entropy, which are informed by global disorder arising from all motions and energy states, we derive a localized measure of entropy to quantify atom-packing DoF in a pore-point-specific manner. This methodological novelty is not merely of theoretical interest; it is motivated by the fact that the performance of machine learning algorithms that predict disease hot-spots in NaVChs largely depends on capturing conserved patterns [37], [38] which encode how geometric and hydropathic characteristics vary across the spatial extent of the NaVCh structure [37], [39]. We report universal trends in dimensionality reduction, atom-packing entropy variation along the pore, and symmetry breaking of hydropathic dipole fields across the NaVCh superfamily, based on analysis of 121 experimentally resolved full-atom structures from both prokaryotic and eukaryotic sources. At the single-molecule level, we characterize mutation clustering relative to the PD/VSD interface in NaV1.7 and establish a transparent machine-learning framework that identifies structural locations where steepening of the sandpile slope may correlate with human pain phenotypes.

## 2 Methods

### Setting the scene

**Fig. 1:**
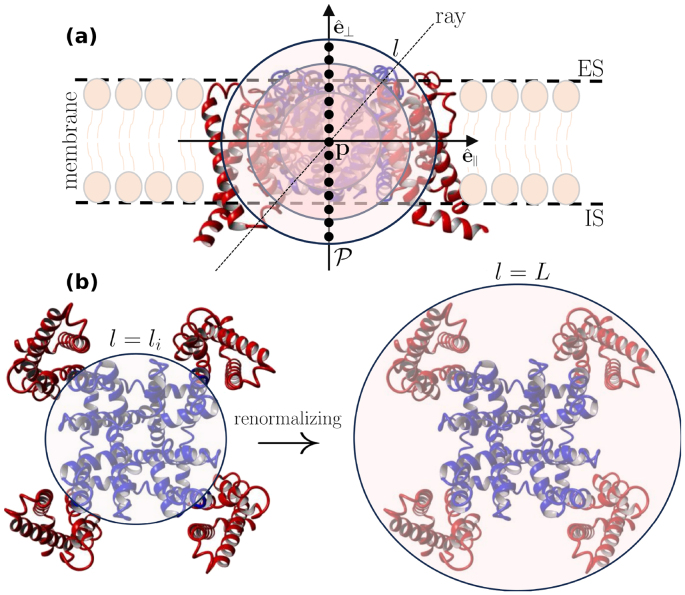
**Illustrative summary of the renormalization group procedure for a voltage-gated sodium channel**. **(a)**, The illustrated molecular side view corresponds to a pre-open NaVAb molecule (PDB code: 3rvy). The pore domain (PD) and voltage sensor domains (VSDs) are illustrated in blue and red, respectively. ${\hat{e}}_{∥}$ and ${\hat{e}}_{⊥}$ are the membrane-parallel and membrane-perpendicular unit vectors, respectively. We introduce consecutive pore points, p∈P (SI Eq. (S1)), forming a path through the NaVAb pore. Each pore point serves as the center of an ensemble of nested balls, each of them characterized by a radius l [Å] (Eq. (1)). An infinite number of radial paths (rays) emanate from each pore point in all directions, making analysis of the atomic environment along individual paths impractical. The renormalization group procedure solves this problem by collapsing radial paths into l, rendering atomic environment properties inherently dependent on l. *Coarse-graining*[41] then enables the computation of relevant scaling exponent. For example, substituting ln⁡(n) for O in Eq. (30), returns via (31) the order parameter scaling exponent, $\Lambda =\pm \frac{\xi d_{f}}{l}$ (SI Eq. (S15)). **(b)**, Top view, from the extracellular side (ES). $l_{i}$ [Å] represents the characteristic size of the PD, also marking the inflection point of the cumulative atom number ((5), (6)). L [Å] is the radius of the smallest ball that contains the entire molecule (SI Eq. (S4)). IS stands for intracellular side.

NaVChs are modular biomolecules organized around a central pore axis that defines the ion conduction pathway (Fig. 1(a)). Understanding their functional architecture therefore requires analyzing molecular dynamics trajectories across multiple temporal and spatial scales. A prerequisite for such an analysis is that the NaVCh adopts a sufficiently long-lived or frequently revisited metastable state.

We focus on observables that remain invariant under transformations of NaVCh size (or scale), conformational state, and subtype. Although this perspective may seem unconventional to molecular biologists accustomed to annotating channel regions based on structural and physicochemical distinctions, our framework complements that detailed view by revealing how apparently disparate features can be unified through mathematically tractable design principles.

To illustrate the approach, imagine a probe designed for the molecular environment. Choose a coordinate of interest, denoted p, representing a biophysically meaningful location within the molecule. From p, initiate a trajectory by extending a radial path (ray) outward toward the molecular ‘rim’ (Fig. 1(a) and (b)). By evaluating all such paths, we can characterize how the local environment around p changes as a function of radial distance. At each step along a given path, the probe records measurement data; once a predefined distance is reached, we return to p, select a new radial path, and repeat the process. Naturally, p corresponds to a pore point, a coordinate along which ion transport dynamics unfold.

However, because infinitely many rays pass through any pore point, this probe protocol becomes infeasible: it enters an endless loop over all possible radial directions, preventing completion of the measurement set for any given p. To avoid this impasse while still obtaining sufficiently informative data, we introduce a renormalization group (RG) technique [36], [40] operating directly in molecular space. The RG procedure collapses the infinitely many rays into a single parameter, l [Å] (Fig. 1(a) and (b)), making all probe measurements explicit functions of radial distance. This coarse-graining operation [41] provides an unbiased representation of information across all radial directions while eliminating path-specific details.

The objective of this RG approach is to substantially reduce atom-packing degrees of freedom (DoF) while preserving the essential molecular physics. A successful RG implementation should yield only a small number of parameters defining a low-dimensional representation of NaVCh functional architecture. This, in turn, allows us to derive, rather than assume, the machine-learning features used to classify variants according to their structural location [37], [38], [39], [42], which is a crucial advantage, as such methods perform best when the underlying feature space admits a low-dimensional parameterization.

**Fig. 2:**
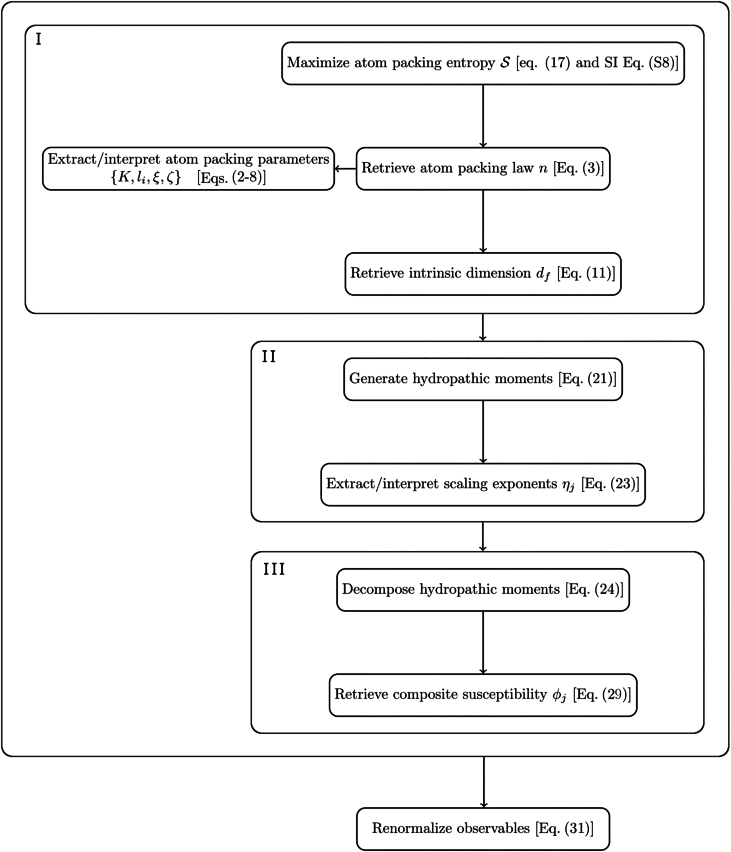
**Analytical procedures guide**. Our starting point is to retrieve the atom-packing law and its associated intrinsic dimensionality measure via an appropriate maximum entropy procedure (upper box (I)). To understand the physicochemical forces that stabilize atom-packing patterns, we study the scaling behavior of hydropathic moments (upper box (II)). To derive machine learning features, we decompose a hydropathic moment into its hydrophobic and hydrophilic components in order to better understand how the ‘competition’ between oppositely acting physicochemical forces determines a molecule’s susceptibility to environmental and/or genetic perturbations (upper box (III)). For theoretical completeness, we recall that the observables appearing in the upper box adhere to a common renormalization rule (lower box).

To orient the reader for Section 2.1, Fig. 2 provides a logical-flow diagram summarizing the key mathematical operations.

### 2.1 Analytical procedures

#### 2.1.1 Spatial organization principles

We focus on a pore point, $p=({\text{p}}_{x},{\text{p}}_{y},{\text{p}}_{z}\equiv {\text{p}}_{⊥})\in P$ (SI Eq. (S1) and Fig. 1(a), and introduce the open ball:

(1) $$\begin{array}{l} B(p,l)=\{(x,y,z)\in R^{3}\;\;|\;\;\text{r}<l\},\;\text{r}=\|\overset{=\vec{r}}{\overbrace{p-(x,y,z)}}\|\;[\text{Å}], \end{array}$$

where l [Å] is the probe radius and $\vec{r}$ is the vector from p to a candidate atom coordinate, (x,y,z). ‖⋅‖ is the Euclidean norm. ∂B={(x,y,z)|r=l} denotes the spherical surface of B. In a structural biology context, ∂B is called an *interface*.

Mathematical functions and associated parameters appearing henceforth depend on (p,l) and p, respectively. For clarity, this dependence is omitted.

##### 2.1.1.1 Packing of atoms

The number of atoms residing on ∂B is continuously approximated by the following generalized logistic differential equation [34], [43]:

(2) $$\begin{array}{l} \partial n=\left(\overset{\text{repulsion term}}{\overbrace{n/\zeta }}-\underset{\text{stabilization term}}{\underbrace{n^{\nu +1}/(\zeta K^{\nu })}}\right)\partial l\;\text{[atom]}\;\text{for}\;n_{0}>0,\\ \text{with}\;K>0\;\text{[atom]}\;\text{and}\;\nu =\frac{\zeta }{\xi },\;\zeta >0\;[\text{Å}],\;\xi >0\;[\text{Å}]. \end{array}$$

n is the number of atoms residing inside B. ζ represents the effective range over which atoms push each other apart (i.e., repel each other). On the other hand, ξ represents the effective range over which atoms pull each other together (i.e., attract each other). $n_{0}=n(l_{0})$ is the atom-packing initial condition realized for some initial l-value, $l_{0}$. This means $n_{0}$ is the number of atoms residing in the initial ball, $B_{0}=B{|}_{l=l_{0}}$. K [atom] is the carrying capacity of the NaVCh structure. It delimits the number of atoms residing inside B for l→∞ (see also (3)), thereby setting an upper bound on the number of atoms the structure can accommodate.

We propose an effective phenomenology supporting (2) based on the interplay between hydrophobic attraction and hydrophilic repulsion [44]. The nonlinear term on the right hand side (RHS) of (2) accounts for attractive, hydrophobicity-driven atomic interactions stabilizing an n-cluster. Namely, it reflects the tendency of hydrophobic constituents to ‘hide’ inside B. The term $n^{\nu +1}$ indicates that any group of ν+1 atoms can form an interaction subnetwork. Since no spatial constraints apply to the formation of such a group, $n^{\nu +1}$ also accounts for long-range interatomic interactions. Hence, ν=ζ/ξ explains the emergence of long-range interactions through bond length adjustment driven by atomic repulsivity.

Accordingly, the linear term appearing in the RHS of Eq. (2) accounts for repulsive, hydrophilicity-driven interactions pushing atoms toward ∂B. This is attributed to water structuring effects that increase the energy required for efficient packing of atoms, according to the thermodynamic self-assembly principles outlined in [45]. Additionally, ζ incorporates the Pauli exclusion principle, as discussed in Ref. [46].

##### 2.1.1.2 The characteristic size of a pore domain

Solving (2) for n gives:

(3) $$\begin{array}{l} n=K{\left(1+\nu \frac{\varepsilon }{\varepsilon_{i}}\right)}^{-1/\nu }\;\text{[atom]},\;n_{l\to \infty }=K\;\text{[atom]}, \end{array}$$

where

(4) $$\begin{array}{l} \varepsilon =\varepsilon_{0}\text{exp}\left(-l/\xi \right)\;[\text{kcal/atom}]\\ \text{with}\;\varepsilon_{i}=\varepsilon {|}_{l=l_{i}}=\varepsilon_{0}\text{exp}\left(-l_{i}/\xi \right)\;[\text{kcal/atom}],\\ \;l_{i}=\xi \text{ln}\left(\frac{{\left(n_{0}/K\right)}^{-\nu }-1}{\nu }\right)+l_{0}\;[\text{Å}]. \end{array}$$

ε imposes an upper bound on the average unsigned hydropathic energy of an individual atom. On the basis of simplicity, $\varepsilon_{0}$ [kcal/atom] is chosen to be a constant. Accordingly, $n_{0}\varepsilon_{0}$ [kcal] delimits the absolute hydropathic energy stored in the initial ball, $B_{0}$. 1/ξ [Å ^-1^] can then be interpreted as the rate at which hydrophobic energy surpluses, stored inside $B_{0}$, are consumed in useful (stabilizing) interactions as the n-cluster size increases. We note that this interpretation is simply a reformulation of our initial understanding about 1/ξ deduced from (2). Specifically, decreasing ξ increases ν, thereby increasing the likelihood of distant atom interactions, reflecting the faster depletion of hydrophobic energy surpluses into stabilizing, potentially long-range bonds.

We do not seek a biophysical interpretation for the logarithmic argument, $\frac{{\left(n_{0}/K\right)}^{-\nu }-1}{\nu }$. Instead, we focus on $l_{i}$.

We emphasize that $l_{i}$ marks the l-value for which the second-order derivative of n with respect to l, i.e., the curvature of n along the radial direction,

(5) $$\begin{array}{l} \frac{\partial^{2}n}{\partial l^{2}}=\frac{n}{\xi^{2}}\frac{\left({\left(\frac{\varepsilon }{\varepsilon_{i}}\right)}^{-1}-1\right)}{{\left({\left(\frac{\varepsilon }{\varepsilon_{i}}\right)}^{-1}+\nu \right)}^{2}}\;[\text{atom}/{\text{Å}}^{2}], \end{array}$$

becomes zero. Stated differently, $l_{i}$ is an inflection point of n. It follows that for $l=l_{i}$, (2) is globally maximized, i.e.,:

(6) $$\begin{array}{l} \underset{l}{\text{max}}\left\{\frac{\partial n}{\partial l}\right\}=\frac{\partial n}{\partial l}{|}_{l=l_{i}}. \end{array}$$

The biophysical significance of (6) becomes apparent when considering empirical evidence suggesting that $\partial B_{i}=\partial B{|}_{l=l_{i}}$ serves as an approximation of the interface mediating the structural transition from the PD to the VSDs [34], [35]. Accordingly, we treat $l_{i}$ as the characteristic length scale of the PD sub-architecture, i.e., the maximum size at which the PD retains its essential functional and structural characteristics, independent of VSD influence (for an illustration see Fig. 1(b), left). Namely, beyond $l_{i}$ (i.e., for $l>l_{i}$), the coupling interactions between a pore module and a radially succeeding voltage sensor cannot be neglected, driving the structural transition from the former toward the latter.

The irregular cylindrical surface emerging from the dense arrangement of $B_{i}$ balls along P is parameterized by:

(7) $$\begin{array}{l} \partial \underset{p}{⋃}B_{i}. \end{array}$$

(7) serves as the characteristic cover for the PD, in the sense that it incorporates essential structural elements of the PD sub-architecture.

##### 2.1.1.3 Mean-field conditioning of the atomic environment

The length scale

(8) $$\begin{array}{l} l_{i,\text{b}}:=l_{i}-\xi \text{ln}\left(\frac{2^{\nu }-1}{\nu }\right)\;[\text{Å}], \end{array}$$

denotes the l-value for which the atom-packing condition n/K=0.5 is satisfied, meaning exactly half of the K atoms are residing inside $B_{i,\text{b}}=B{|}_{l=l_{i,\text{b}}}$. ‘b’ stands here for ‘bound’, since $l_{i,\text{b}}$ imposes an upper and lower bound on $l_{i}$, depending on whether the attractive or repulsive interaction range prevails, i.e., whether ν<1 or ν>1, respectively. Accordingly, the negative and positive sign of $\text{ln}\left(\frac{2^{\nu }-1}{\nu }\right)$ decides whether the length scale bound applies from below or from above, i.e., whether $l_{i,\text{b}}>l_{i}$ or $l_{i,\text{b}}<l_{i}$, respectively.

If the attractive interaction range equals the repulsive interaction range (i.e., ν=1), then $\text{ln}\left(\frac{2^{\nu }-1}{\nu }\right)=0$ and $l_{i,\text{b}}=l_{i}$, and (3) becomes isomorphic with the Fermi-Dirac (i.e., logistic) distribution. Hydrophobicity-driven attraction and hydrophilicity-driven repulsion are then delicately balanced, imposing a mean-field conditioning on the atomic environment around a pore point. Across an evolutionary time scale, ν=1 promotes isotropic space exploration in the PD and the VSDs, as an equal number of atoms is distributed in the PD and the VSDs (i.e., half of the K atoms are found inside $B_{i}=B_{i,\text{b}}$ and the other half outside of it). Generally, this favors the emergence of globular-like molecular shapes, as demonstrated in Ref. [46], which, in the context of this study, is driven by a roughly equal and uniform allocation of masses inside and outside $B_{i}$.

##### 2.1.1.4 Unit mass fractal dimension

Expressing (2) in terms of (3), reveals that packing of atoms around a pore point satisfies the self-similarity relationship:

(9) $$\begin{array}{l} \frac{1}{K}\frac{\partial n}{\partial l}=\frac{\varepsilon }{\varepsilon_{i}\xi }{\left(\frac{n}{K}\right)}^{\nu +1}\;[{\text{Å}}^{-1}], \end{array}$$

which implies that:

(10) $$\left\{ \begin{array}{l} \int_{l_{0}}^{L}\text{d}l\;\frac{\partial n}{\partial l}\to K\;[\text{atom}]\;\left(a\right)\\ \int_{l_{0}}^{L}\text{d}l\;{\left(\frac{n}{K}\right)}^{\nu +1}\varepsilon \to \varepsilon_{i}\xi \;[\text{kcal}\times \text{Å}/\text{atom}]\;\left(b\right) \end{array}\right.\text{for}\;l_{0}\to 0\;\text{and}\;L\to \infty ,$$

where $B_{L}=B{|}_{l=L}$ is the smallest ball that accommodates all NaVCh structure atoms (SI Eq. (S4) and Fig. 1(b) right part).

(10) introduces two distinct yet interrelated constraints. Specifically, (10)(a) and (10)(b) ensure that even if the molecular radial size, s [Å] (SI Eq. (S5)), becomes infinitely large, the total number of atoms cannot exceed K, and the hydropathic interaction energy per atom converges to $\varepsilon_{i}\xi$ [kcal×Å /atom], respectively.

The intrinsic dimension (i.e., the unit mass fractal dimension [47]) of an n-cluster is continuously measured with:

(11) $$\begin{array}{l} d_{f}=\frac{\partial \text{ln}(n)}{\partial \text{ln}(l)}=\frac{l}{\xi \left({\left(\frac{\varepsilon }{\varepsilon_{i}}\right)}^{-1}+\nu \right)}. \end{array}$$

(11) describes how ‘intensively’ (or ‘compactly’, as discussed in Ref. [48]) n atoms fill space inside B. Generally, the fraction of empty space inside B increases with decreasing $d_{f}$.

#### 2.1.2 Atom-packing entropy

Let us now consider the following discretization of l:

(12) $$\begin{array}{l} l_{\alpha }=\bar{R}+\frac{\alpha }{N_{\alpha }}s\;[\text{Å}],\;\alpha =1,2,\ldots ,N_{\alpha }\in Z_{>0}, \end{array}$$

where α is a scale index [34], [43]. $N_{\alpha }$ is the total number of scale iterations, determining the resolution of the discretization. $\bar{R}$ [Å] is the distance separating p from its nearest-neighbor atom, while s [Å] remains the molecular radial size (first considered below (10) and given by SI Eq. (S5)). Note that $B{|}_{l=\bar{R}}$ is devoid of atoms.

The probability that an atom resides inside $B_{\alpha }=B{|}_{l=l_{\alpha }}$ is given by:

(13) $$\begin{array}{l} p_{\alpha }=\frac{{\left(1+\nu \frac{\varepsilon_{\alpha }}{\varepsilon_{i}}\right)}^{-1/\nu }}{Z_{p}}=\frac{n_{\alpha }/K}{Z_{p}},\;Z_{p}=\sum_{\alpha }n_{\alpha }/K, \end{array}$$

where ${\left(1+\nu x\right)}^{-1/\nu }$, x≥0, is identified with the survival function of a Pareto Type-II distribution [49]. $Z_{p}$ is the partition sum guaranteeing that $\sum_{\alpha }p_{\alpha }=1$.

The probability that (ν+1)-atoms reside inside $B_{\alpha }$ is given by the Escort probability [50]:

(14) $$\begin{array}{l} \pi_{\alpha }=\frac{p_{\alpha }^{\nu +1}}{Z_{\pi }}=\frac{{\left(n_{\alpha }/K\right)}^{\nu +1}}{Z_{\pi }Z_{p}^{\nu +1}},\;Z_{\pi }=\sum_{\alpha }p_{\alpha }^{\nu +1}, \end{array}$$

where $Z_{\pi }$ is the corresponding partition sum. π’s biophysical importance stands out when we consider the following finite-size corrected version of (10(b)):

(15) $$\begin{array}{l} C\sum_{\alpha }\Delta l_{\alpha }\pi_{\alpha }\varepsilon_{\alpha }=\varepsilon_{i}\overset{=:\Xi \;\left[\text{Å}\right]}{\overbrace{\left(\xi +c_{s}/s+c_{\alpha }/N_{\alpha }\right)}}\;[\text{kcal}\times \text{Å}/\text{atom}]\\ \Delta l_{\alpha }=l_{\alpha +1}-l_{\alpha }\;[\text{Å}],\;C=Z_{p}^{\nu +1}Z_{\pi }, \end{array}$$

where Ξ is a finite-size corrected version of ξ. $\left|c_{s}\right|$ [Å ^2^] and $\left|c_{\alpha }\right|$ [Å] account for interfacial and radial geometric distortions due to s’s and $N_{\alpha }$’s finiteness, respectively. Note that $\Delta l_{\alpha }$ is constant. C is a normalization factor whose specific value is irrelevant.

Crucially, for $N_{\alpha }\to \infty$, the constraint (10)(b) can be recovered:

(16) $$\begin{array}{l} C\sum_{\alpha }\Delta l_{\alpha }\pi_{\alpha }\varepsilon_{\alpha }\overset{N_{\alpha }\to \infty }{\to }\int_{l_{0}}^{L}\text{d}l\;{\left(\frac{n}{K}\right)}^{\nu +1}\varepsilon \overset{l_{0}\to 0,L\to \infty }{\to }\varepsilon_{i}\xi \;[\text{kcal}\times \text{Å}/\text{atom}]. \end{array}$$

An entropy functional whose maximization explicitly incorporates (13), (14), (15) is given by:

(17) $$\begin{array}{l} S=k\frac{1-\sum_{\alpha }p_{\alpha }^{q}}{q-1}\;[\text{kcal}/(\Theta \times \text{atom})],\;q=1+\nu , \end{array}$$

where q is known as the nonextensivity index [51], [52] and k [kcal/(Θ× atom)] is the equivalent of Boltzmann’s constant. Note that [Θ] is the physical unit at which the temperature of the current pore point is measured (SI 1.4).

From an information theoretical viewpoint, S can be trivially interpreted as the average amount of surprise (or ‘unexpectedness’) associated with determining how many atoms reside inside $B_{\alpha }$.

From an evolutionary perspective, S illustrates how the competition between hydrophobicity-driven attraction and hydrophilicity-driven repulsion determines the informational content of the atomic environment around a pore point.

Local scarcity of hydrophilicity can attenuate associated repulsion effects (i.e., ζ→0), promoting the formation of a hydrophobic core. S then approaches the following upper bound:

(18) $$\begin{array}{l} S_{q\to 1}=-k\sum_{\alpha }p_{\alpha ,\nu \to 0}\text{ln}(p_{\alpha ,\nu \to 0})\;[\text{kcal}/(\Theta \times \text{atom})], \end{array}$$

where $p_{\alpha ,\zeta \to 0}\propto \frac{n_{\zeta \to 0}}{K}$ is a common exponential (SI Eqs. (S6) and (S10)). Generally, the limit ζ→0 does not necessitate a scarcity of hydrophilic components but it can arise from screening, where the surroundings attenuate interatomic repulsivity inside B, causing the effective interaction range to collapse, even with a finite concentration of repulsive particles still present.

Maximizing S toward $S_{q\to 1}$ expands the NaVCh configuration space volume, since the amount of water required to solvate a hydrophobic atom group inside B is typically less than that required for solvating n individually dispersed hydrophobic atoms. The remaining, non-solvating water molecules can then uncoordinatedly engage in hydrogen bond interactions with NaVCh constituents on ∂B, thereby increasing atom-packing DoF. Simultaneously, we notice that the intrinsic dimension of the PD,

(19) $$\begin{array}{l} d_{f}{|}_{l_{i}}\overset{\text{(11)}}{=}\frac{l_{i}}{\xi q}, \end{array}$$

is decreased, since

(20) $$\begin{array}{l} \frac{l_{i}}{\xi q}\overset{\text{(4)}}{=}\frac{1}{q}\left(\text{ln}\left(\frac{{\left(K/n_{0}\right)}^{q-1}-1}{q-1}\right)+\frac{l_{0}}{\xi }\right)\overset{\zeta \to 0}{\to }\ln(\ln(K/n_{0}))+l_{0}/\xi \end{array}$$

is monotonically and positively correlated with ζ for fixed parameter values $\left\{l_{0},n_{0},K,\xi \right\}$ (SI Fig. S1 inset).

(20) illustrates how the PD sub-architecture can mitigate the potentially disorganizing effect of excess (‘redundant’) atom-packing DoF by lowering its intrinsic dimension.

#### 2.1.3 Thermostability

##### 2.1.3.1 Empirical measure

The thermostability of an n-cluster is probed with the hydropathic moments toolbox [34], [35], [43], [53], [54], [55], [56]:

(21) $$\begin{array}{l} \;\int_{B}\text{d}\vec{r}\sum_{a\in A}\delta (\vec{r}-{\vec{r}}_{\text{a}}){\left({\vec{r}}_{\text{a}}\right)}^{j}w_{\text{a},\iota }\\ \;=\left\{ \begin{array}{l} h_{j},\;j=2k,\;k\in Z_{\geq 0}\\ {\vec{h}}_{j}=\overset{={\vec{h}}_{j,∥}}{\overbrace{h_{j,∥}{\hat{e}}_{∥}}}+\overset{={\vec{h}}_{j,⊥}}{\overbrace{h_{j,⊥}{\hat{e}}_{⊥}}},\;j=2k+1 \end{array}\right.\;\text{[kcal}\times {\text{Å}}^{j}\text{]}, \end{array}$$

where δ(⋅) is a generally defined Dirac delta function, a∈A is an atom coordinate with ${\vec{r}}_{\text{a}}$ denoting the vector from p to a, and $w_{\text{a},\iota }=w_{\text{a}}+\iota$ [kcal], $\iota ∼N(0,\sigma_{\iota }=1\text{e}-03)$, are noise-perturbed hydropathic weights originating from the Kapcha&Rossky atomic hydropathic scale [57]. Noise accounts here for randomly occurring water density fluctuations.

Note that ${\vec{h}}_{j,∥}$ and ${\vec{h}}_{j,⊥}$ act parallel and perpendicular, respectively, to the membrane, surface with ${\hat{e}}_{∥}$ and ${\hat{e}}_{⊥}$ being the corresponding unit vectors (SI S1.2.3). Conventionally, ${\hat{e}}_{⊥}$ points toward the extracellular side (ES).

The subscript ‘j’ indicates the moment order determining the dimension of the space within which water-mediated interactions are probed. j-parity determines whether we probe inertia (j=2k) or conductivity (j=2k+1) constraints, concerning rotational and translational atom-packing DoF expressed around and along the pore point path, P, as detailed in SI 1.5.1–2, respectively.

##### 2.1.3.2 Theoretical model

Normalizing $h_{j}$ over n yields the n-cluster hydropathic density [53], [54], [55], [56] whose oscillatory behavior is described by:

(22) $$\begin{array}{l} \frac{h_{j}}{n}=A_{j}\cos(f_{j})\;[\text{kcal}\times {\text{Å}}^{j}/\text{atom}]\\ \text{with}\;A_{j}=(1+\kappa_{j}\xi /s)\varepsilon l^{j}\overset{\text{(4)}}{=}(1+\kappa_{j}\xi /s)\varepsilon_{0}\exp(-l/\xi )l^{j}, \end{array}$$

where $f_{j}$ determines the sign changing behavior of $h_{j}$ and $A_{j}$ envelopes the underlying wave packet. The factor $(1+\kappa_{j}\xi /s)>0$ introduces a finite-size correction, with $|\kappa_{j}|\xi$ [Å] being the adsorption bond length. $\kappa_{j}$ is dimensionless and purely phenomenological, as it can only be deduced by fitting $A_{j}\cos(f_{j})$ to experimental $h_{j}/n$-traces. It generally describes the degree of adhesion of atoms on ∂B in response to interfacial tensions, with its sign indicating whether these tensions lead to an increase or decrease in $\varepsilon_{0}$.

Notably, $A_{j}$ preserves the general hydropathic energy form used in Refs. [58], [59], [60]. Moreover, consistent with Eq. (4), for j=0, we verify that $A_{0}\overset{s\to \infty }{\to }\varepsilon \geq |h_{0}|/n$, illustrating that ε serves as an upper bound for the average unsigned hydropathic energy of an individual atom.

##### 2.1.3.3 Scaling of hydropathic dipole field amplitude

Hydropathic energy wave packet self-modulation effects persisting over sufficiently large l-intervals covering the PD and the VSDs are described by [34], [35]:

(23) $$\begin{array}{l} \;|h_{j}|\leq nA_{j}\propto \left\{ \begin{array}{l} l^{\eta_{j,<}},\;l\in (l_{0},l_{i}]\\ l^{\eta_{j,>}},\;l\in (l_{i},l_{>}] \end{array}\right.\;, \end{array}$$

where $l_{>}$ [Å] denotes the l-value for which the n-curvature (Eq. (5)) is negatively maximized.

$(\eta_{j,<},\eta_{j,>})\in R^{2}$ are the corresponding scaling exponents. $\left|\eta_{j,<}\right|$ and $\left|\eta_{j,>}\right|$ set an upper bound on how ‘intensively’ j-th-order dipole-dipole hydropathic interactions can fill space in the PD and the VSDs, respectively.

The sign of $\eta_{j}$ indicates the direction of hydropathic interaction network intensification, either inward (i.e., $\eta_{j}<0$) or outward (i.e., $\eta_{j}\;>\;0$).

#### 2.1.4 Mutational robustness

##### 2.1.4.1 Decomposability

Sign-changes of $h_{j}$ mark n-clusters of diminished thermostability. Mechanofunctional properties of these n-clusters are expected to be sensitive to perturbations.

To understand how the multiscale competition between hydrophobic attraction and hydrophilic repulsion can lead to varying thermostability and emergent mechanofunctional properties, we consider the following decomposition ansatz:

(24) $$\begin{array}{l} h_{j}=h_{j,+}+h_{j,-}\;\;\text{for}\;\;l>l_{\text{cutoff}}\\ \text{with}\;\;h_{j,+}>0,\;\;h_{j,-}<0,\;\;n=n_{+}+n_{-},\\ \text{and consider:}\\ h_{j,+}:=h_{j,\text{philic}},\;\;h_{j,-}:=h_{j,\text{phobic}},\;\;n_{+}:=n_{\text{philic}},\;\;n_{-}:=n_{\text{phobic}}\;\;\text{(case A)},\\ \text{or}\\ h_{j,+}:=h_{j,\text{phobic}},\;\;h_{j,-}:=h_{j,\text{philic}},\;\;n_{+}:=n_{\text{phobic}},\;\;n_{-}:=n_{\text{philic}}\;\;\text{(case B)}, \end{array}$$

where $l_{\text{cutoff}}$ is the ansatz cutoff scale.

We verify that for vanishingly small noise, i.e., $\sigma_{\iota }\to 0$ (see (21)), and j=2k, case A holds. Namely, $h_{j,+}=h_{j,\text{philic}}$ and $h_{j,-}=h_{j,\text{phobic}}$ are the contributions of only hydrophilic and hydrophobic atoms, respectively, with $n_{+}=n_{\text{philic}}$, $n_{-}=n_{\text{phobic}}$ and $l_{\text{cutoff}}\to l_{0}$.

Whether case A or B holds for the pair $\left\{h_{2k+1,⊥,\text{philic}},h_{2k+1,⊥,\text{phobic}}\right\}$ requires computational investigation, as there is no general argument supporting this assertion.

Since $h_{j,+}$ and $h_{j,-}$ are cumulative, and thus slowly changing, sign-preserving functions over the l-range, we can straightforwardly compute their continuously varying scaling exponents:

(25) $$\psi_{j,+}=\frac{\partial \text{ln}(h_{j,+})}{\partial \text{ln}(l)}=\frac{\partial h_{j,+}}{\partial l}\frac{l}{h_{j,+}}$$

and

(26) $$\psi_{j,-}=\frac{\partial \text{ln}(-h_{j,-})}{\partial \text{ln}(l)}=\frac{\partial h_{j,-}}{\partial l}\frac{l}{h_{j,-}},$$

respectively.

The physical meaning of $\psi_{j,+}$ and $\psi_{j,-}$ complements that of η. Namely, $\left|\psi_{j,+}\right|$ and $\left|\psi_{j,-}\right|$ account for the intensification of the j-th-order dipole-dipole interaction network stabilizing the subgroups of $n_{+}$ and $n_{-}$ atoms, respectively, along the radial direction.

The signs of $\psi_{j,+}$ and $\psi_{j,-}$ indicate whether the direction of interaction network intensification is inward (if $\psi_{j}<0$) or outward (if $\psi_{j}\;>\;0$).

##### 2.1.4.2 Logarithmic composite susceptibility

The difference between $\psi_{j,-}$ and $\psi_{j,+}$,

(27) $$\Delta \psi_{j}=\psi_{j,+}-\psi_{j,-},$$

illustrates how the interaction networks, stabilizing the subgroups of $n_{+}$ and $n_{-}$ atoms, ‘compete’ to fill space inside B.

We postulate that when $\Delta \psi_{j}=0$, the two networks are in a state of balance, where perturbations affecting the $n_{+}$ and $n_{-}$ subgroups tend to induce oppositely directed ∂B-responses, effectively canceling each other out. On the other hand, $\Delta \psi_{j}\neq 0$ implies an interaction network imbalance, determining also the prevailing direction of a ∂B-response. Accordingly, the sign of $\Delta \psi_{j}$ informs about the direction of perturbation-induced ∂B-responses.

Dividing $\Delta \psi_{j}$ by l defines the following size-normalized measure of interaction network imbalance:

(28) $$I_{j}:=\frac{\Delta \psi_{j}}{l}\;[{\text{Å}}^{-1}],$$

assuming that the interaction network imbalance is uniformly distributed over the radial extent of an n-cluster.

Integrating $I_{j}$ over an l-range, yields the logarithm of the j-th-order composite susceptibility, i.e.,:

(29) $$\begin{array}{l} \phi_{j}:=\text{ln}\left(\chi_{j}\right)=\int_{l_{0}}^{L}\text{d}l\;I_{j}\\ \text{with}\;\chi_{j}:=-\frac{h_{j,+}}{h_{j,-}}, \end{array}$$

with $\chi_{j}$ being identified as the j-th-order composite susceptibility. Generally, $\chi_{j}$ can be interpreted as a descriptor of how internally generated hydrophobicity fields couple with internally generated hydrophilicity fields and vice versa.

Treating $-\frac{h_{j,+}}{h_{j,-}}$ as an effective temperature ratio reveals that $\left|\phi_{j}\right|$ is analogous to a thermostability geodesic distance (SI S1.6.1). Simply put, $\left|\phi_{j}\right|$ quantifies how ‘far’ an n-cluster’s thermostability state is from a state of minimal thermostability (or, maximal sensitivity to perturbations).

We emphasize that although both $\left|\phi_{j}\right|$ and $\left|h_{j}\right|$ qualify as thermostability ‘distance’ measures (since $h_{j}=0\overset{\text{(24)}}{\;⟹\;}\phi_{j}=0$), the geodesic property applies exclusively to $\left|\phi_{j}\right|$. In fact, while $\eta_{j}$ (Eq. (23)) provides information at an envelope level, $\Delta \psi_{j}$ (27) reflects the actual sculpting (i.e., the radial interplay of successive peaks and valleys) of the hydropathic energy wave packet.

On the grounds of consistency, $I_{j}$ is considered to provide equivalent information to $\phi_{j}$ but at an interface level. Namely, $\left|I_{j}\right|$ measures how ‘far’ an ∂n-shell’s thermostability state is from a state of minimal thermostability. We term $I_{j}$ the ‘interfacial coupling strength’.

#### 2.1.5 Renormalizability

The RG flow equation has the general form [61]:

(30) $$\begin{array}{l} \frac{\partial O}{\partial \text{ln}(\tau )}=\Lambda (O),\;\tau =\left\{ \begin{array}{l} \frac{\varepsilon }{\varepsilon_{i}},\;\partial B_{i}\mapsto \partial B\;\;(\text{perturbed from}\;\;\partial B_{i}\;\;\text{onto}\;\;\partial B)\\ \frac{\varepsilon_{i}}{\varepsilon },\;\partial B\mapsto \partial B_{i}\;\;(\text{perturbed from}\;\;\partial B\;\;\text{onto}\;\;\partial B_{i}) \end{array}\right.\;\;\;\;, \end{array}$$

where O:=O(p,l) is a NaVCh structure log-observable (referred to as the *effective coupling* [61]). τ is the normalized hydropathic energy *scale* [61], representing the relative temperature change in response to PD/VSD interface fluctuations (SI Eq. (S13)). Λ describes the dependence of O on τ and is the equivalent of the *beta function* [61]. It represents a continuously varying scaling exponent attaining its critical value precisely at τ=1 (SI 1.7).

The conceptual basis upon which (30) is built, is illustrated in Fig. 1(b) along with its caption.

From (30), we straightforwardly obtain the renormalizability relationship:

(31) $$\begin{array}{l} \frac{\partial O}{\partial \text{ln}(l)}=\pm \frac{l}{\xi }\Lambda (O) \end{array}$$

Eq. (31) establishes that changes in O over a logarithmic radial range (on the left hand side of (31)) can be equivalently obtained by considering changes in O driven by PD-subarchitecture deformations reflected in PD/VSD interface fluctuations (on the right hand side of (31)).

### 2.2 Computational procedures

#### 2.2.1 Structure preparation

NaVCh structures are ‘cleaned’, protonated, and their orientation is fixed, following the procedures outlined in SI S1.2.

#### 2.2.2 Curve fitting and model selection

Candidate n-models were fitted to the dimensionless empirical cumulative atom number:

(32) $$\begin{array}{l} \overset{=N}{\overbrace{\int_{B}\text{d}\vec{r}\sum_{a\in A}\delta (\vec{r}-{\vec{r}}_{\text{a}})}}/N_{\text{total}}, \end{array}$$

where $N_{\text{total}}=\text{card}(A)$ is the total number of atoms found in a ‘clean’, protonated NaVCh structure. Algorithmic implementation details are enclosed in SI 1.11.

## 3 Results

### 3.1 NaVCh spatial organization

We compiled a structural dataset comprising 71 prokaryotic NaVCh structures (subtypes: NaChBac, NaVAb, NaVMs, NaVAe, NaVRh) and 50 eukaryotic structures (subtypes: NaVPas, NaVEe1, NaVEh, NaV1-8) of sufficient resolution (SI S1.1).

#### 3.1.1 Geometry

##### 3.1.1.1 inflection point universality

We report an excellent agreement between the empirical cumulative atom number, N (Eq. (32)), and its theoretical counterpart, n (Eq. (3)), across all 121 structures (Fig. 3(a) and (g), SI Figs. S5-S10(a), S12-S22(a)), supported by small mean absolute fitting errors (SI S2.1). P-values remained inconclusive due to the coarse-grained nature of our model and their sensitivity to noise (SI S2.1).

**Fig. 3:**
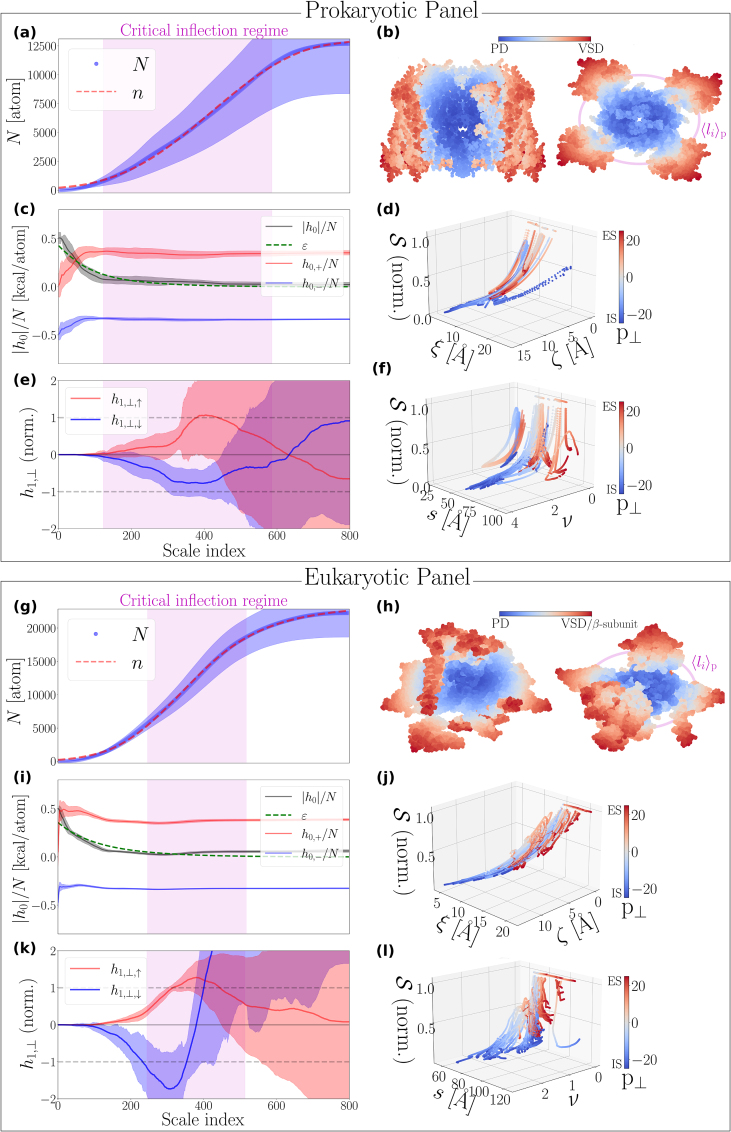
**Spatial organization features of the NaVCh superfamily**. Statistical summary of the spatial-organization features of 71 prokaryotic (subtypes: NaChBac, NaVAb, NaVMs, NaVAe, NaVRh) and 50 eukaryotic (subtypes: NaVPas, NaVEe1, NaV1-8, NaVEh) NaVCh atomic structures (SI Tab. S1), for pore points, p∈P, and scale indices, α=1,2,…,800. **(a)**,**(g)**, Collapsed traces of the empirical, N [atom], and best-fitted theoretical, n [atom], cumulative atom numbers. **(b)** and **(h)** illustrate the prototype NaVAb channel (PDB code: 3rvy) and the human NaV1.7 channel (PDB code: 7w9k), respectively. Atoms are colored according to their PD/VSD ordering score (SI S1.8.1.2), and projected onto a plane perpendicular (left side) and parallel (right side) to the membrane. $\langle l_{i}\rangle_{\text{p}}$ is the mean value (computed along P) of the characteristic size of the pore domain (PD). VSD stands for voltage sensor domain. **(c)**,**(i)**, Collapsed traces of the empirical, $\frac{\left|h_{0}\right|}{n}$ [kcal/atom], and theoretical, ε [kcal/atom], absolute hydropathic energies per atom. $\frac{h_{0,+}}{n}$ is the hydrophilic component, and $\frac{h_{0,-}}{n}$ is the hydrophobic component of $\frac{h_{0}}{n}$, respectively. **(d)**,**(j)**, The interplay between the normalized atom-packing entropy, S, and the pair of interaction ranges, {ξ,ζ}. **(e)**,**(k)**, Collapsed traces of ‘pointing extracellularly (ES)’ and ‘pointing intracellularly (IS)’ instances of the normalized membrane-vertical HDF amplitude, $h_{1,⊥}$ [kcal×Å /atom]. $h_{1,⊥}$ is normalized by $h_{1,⊥}{|}_{l=l_{i}}$. If $h_{1,⊥}{|}_{l=l_{i}}>0$, $h_{1,⊥}$ is labeled as a ‘pointing ES’ (↑) instance; otherwise, ‘pointing IS’ (↓). **(f)**,**(l)**, Nonextensivity of S [kcal/(Θ×atom)], as revealed by its weak and strong dependence on the molecular radial size, s [Å], and the interaction range ratio, $\nu =\frac{\zeta }{\xi }$, respectively. Note that S is normalized by $S_{\text{max}}\equiv S_{q\to 1}$(Eq. (18)). The critical inflection regime marks the interval from which all $l_{i}$ values are drawn. Trace-collapse procedures are described in SI S1.8.1.1.

To appreciate the statistical weight of this finding, we emphasize that our fitting algorithm had to converge approximately 121×690 = 83,490 times, where 121 is the total number of NaVCh structures considered and 690 gives the average number of investigated pore points per NaVCh structure, separated along the pore axis by a sampling distance of 0.1 Å (SI Tab. S2).

These findings indicate that NaVCh atom-packing physics can be compressed into the set of parameters {ζ,ξ,K} ((2), (3)), in a pore-point-specific manner. Key to our understanding is that ξ and ζ are indirect measures of molecular attraction and repulsion, influencing molecular compression and expansion, respectively. Accordingly, the NaVCh molecule is conceptualized as a compressible, fluid-like material, whose mechanofunctional properties vary along its principal pore axis, approximated by the pore point path P. In turn, this implies that the atomic environment around each pore point admits a distinct statistical mechanical description or, equivalently, admits a local temperature linked to the hydropathic energy level of the PD levels (SI S1.4).

The formation of interfacial geometries in compressible materials requires the spontaneous cancellation of interfacial tensions, manifested as minima in the underlying interfacial free energy [62], a principle that also applies to the spatial organization of NaVChs. Structurally, it gives rise to the PD/VSD interface, denoted $\partial B_{i}$ (Eq. (6)), mediating two qualitatively different – in terms of their thermostability character – atomic environments. This insight enables the structural annotation of atoms, determining whether they belong to the PD or the VSDs, as n/K serves as an order parameter distinguishing between two coexisting material phases (Fig. 3(b) and (h), SI Figs. S5-S10(b), S12-S22(b)).

Under mean-field conditions (when ξ=ζ, see below Eq. (8)), n/K admits the standard critical exponent, β=0.5 (SI Eqs. (S15) and (S16)). Moreover, it can be readily shown that β is related to other standard critical exponents through a generalized version of Widom’s scaling law – whose standard form can be recovered when the characteristic renormalization scale matches the correlation length (represented here by $l_{i}$ and ξ, respectively) (SI S1.6.4).

Comparing the $l_{i}/s$-distributions (with s the radial size of the molecule) for prokaryotic and eukaryotic channels shows that, in eukaryotes, the distribution peaks at ≈0.5, whereas in prokaryotes it is broader with multiple peaks (SI Fig. S2(c) vs. (d)), consistent with the greater assembly heterogeneity of the prokaryotic dataset along the pore (e.g., arising from C-terminal extensions (SI Fig. S8)). In both cases, the $l_{i}/s$-ratio remains bounded within [0.25,0.75] guaranteeing that the structural transition occurs within well-defined radial bounds, as also supported by the clear sigmoid profiles exhibited by N (and n) in Fig. 3(a) and (g).

##### 3.1.1.2 Pore domain intrinsic dimensionality

**Fig. 4:**
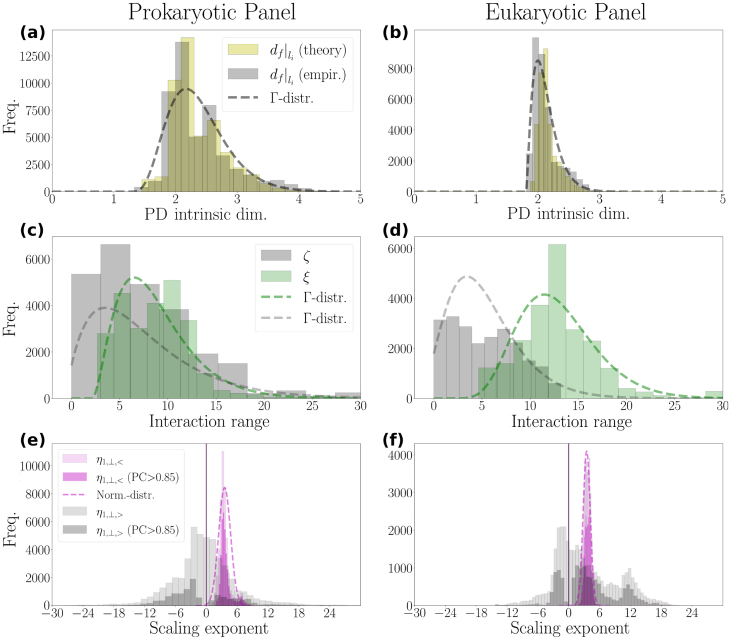
**Intrinsic dimensions, scaling exponents, and attractive-vs.-repulsive interaction range statistics**. Statistical compilation of the pore domain (PD) intrinsic-dimension and interaction-range characteristics for 71 prokaryotic (subtypes: NaChBac, NaVAb, NaVMs, NaVAe, NaVRh) and 50 eukaryotic (subtypes: NaVPas, NaVEe1, NaV1-8, NaVEh) NaVCh atomic structures (SI Tab. S1). **(a)**,**(b)**, Histograms of the empirical and theoretical measures of the PD intrinsic dimension. The Γ-distribution mean values of the empirical intrinsic dimension measure are 2.4 and 2.1, with corresponding standard deviations of 0.5 and 0.2, for prokaryotic and eukaryotic PDs, respectively. **(c)**,**(d)**, Histograms of the attractive and repulsive interaction ranges given by ζ [Å] and ξ [Å], respectively. The Γ-distribution mean values are 8.8 Å and 12.9 Å for ξ, and 7.5 Å and 5.2 Å for ζ, in prokaryotes and eukaryotes, respectively. **(e)**,**(f)**, Histograms of the scaling exponents of the membrane-perpendicular dipole-field component, $h_{1,⊥}$. The exponents $\eta_{1,⊥,<}$ and $\eta_{1,⊥,>}$ account for the scaling behavior of $h_{1,⊥}$ over l-intervals covering the PD and the voltage sensor domains, respectively (Eq. (23)). They are computed according to procedures described in SI S1.8.3. PC stands for Pearson correlation coefficient.

Dimensionality analysis reveals that prokaryotic and eukaryotic PDs exhibit a preference for ‘flatness’, as their intrinsic dimensions (Eq. (19)) are, on average, values only slightly greater than 2 (Fig. 4(a) and (b)).

By tuning the intrinsic dimension of the PD near 2, PD sub-architecture is effectively mapped onto consecutive membrane-parallel planes, with roughness reciprocally related to $\left|d_{f}{|}_{l_{i}}-2\right|$, favoring a smooth cylindrical shape. According to Eq. (20), this reflects a self-regulating mechanism that preserves PD structural and functional integrity by compensating for excess atom-packing DoF generated by a tightened atomic environment arising either from hydrophobic surpluses or attenuated repulsive forces, both of which drive ζ→0: with ξ held well above zero, decreasing ζ reduces interatomic distances and counteracts the expansion of configuration-space volume caused by surplus atom-packing DoF. Lowering the intrinsic dimension therefore increases spatial-organization efficiency in the PD by favoring nearly water-free, planar configurations.

Eukaryotic PDs exhibit greater efficiency in spatial organization, as evidenced by their smaller mean and standard deviation values for intrinsic dimensions compared to prokaryotic PDs (see Fig. 4 caption). This finding is consistent with the general trends reported in Ref. [63], where lower intrinsic dimensions were found to correlate with higher organismal complexity.

#### 3.1.2 Atom-packing energy and entropy

##### 3.1.2.1 An exponentially decaying hydrophobic ‘force’ stabilizes the structure

As illustrated in Fig. 3(c) and (i), a decrease in n-cluster size implies an increase in its average hydrophobicity, $h_{0,-}/N$ ((22), (24)). Specifically, $h_{0,-}/N$ converges toward −0.5 kcal for $l\to l_{0}$ (decreasing scale index), denoting the hydropathic score assigned to an individual hydrophobic atom based on the Kapcha&Rossky hydropathic scale [57]. In contrast, the n-cluster average hydrophilicity, $h_{0,+}/N$ ((22), (24)), tends to vanish for $l\to l_{0}$ (Fig. 3(c) and (i)).

This trend showcases an entropically favorable arrangement in which hydrophobicities ‘hide’ inside B rather than being exposed on its surface, ∂B. As hydrophobic constituents fill the available space inside B, hydrophilic ones are displaced outward, preferentially settling near ∂B. This redistribution creates an initial surplus of hydrophobic energy available to pore-lining constituents, causing the experimentally observed absolute hydropathic energy per atom, $|h_{0}|/N$, to peak as $l\to l_{0}$ (Fig. 3(c) and (i); SI Figs. S5–S10(c), S12–S22(c)).

However, as the n-cluster size grows (increasing scale index), the initial hydrophobic energy surplus is exponentially depleted at a rate governed by ξ and converted into stabilizing interactions that bind the nested B’s together. Intriguingly, this suggests that interfacial adsorption effects do not substantially distort atom-packing conditions (Eq. (22)). In turn, this implies that NaVChs have evolved near a thermodynamic state in which they mimic the spatial organizational traits of larger, bulkier systems. This maximizes NaVCh mechanofunctional efficiency by allowing perturbations to be processed nearly adiabatically (SI S1.6.1). Our analysis supports this intuition, as shown by the good agreement between $|h_{0}|/N$ and ε [kcal/atom] (Fig. 3(c) and (i), SI Figs. S5–S10(c) and S12–S22(c)), where ε is the theoretically predicted absolute hydropathic energy per atom (Eq. (4)).

Evidently, if the repulsive interaction range ζ grows too large, structural integrity could be compromised due to excessive interatomic spacing. To avoid this, the distribution of ζ is located closer to zero than that of ξ (Fig. 4(c) and (d)). Also, in contrast to ξ, ζ has a smaller mean value for eukaryotic NaVChs compared to prokaryotic NaVChs (Fig. 4 caption), supporting the idea that eukaryotic NaVChs, despite being more diverse – both structurally and functionally –, are spatially organized in a more efficient manner. Notably, the mean value of ξ is approximately 8.8 Å and 12.9 Å in prokaryotic and eukaryotic NaVChs, respectively (Fig. 4(c) and (d)). These values are in good agreement with the typical ∼10 Å decay-length readout commonly associated with measuring the exponentially decaying amplitude of hydropathic interaction potentials between molecular surfaces [58], [59], [60], which are explicitly represented here by the interface geometry ∂B.

##### 3.1.2.2 Atom-packing entropy variation along the pore

Moving from the IS to the ES along P, we observe that the atom-packing entropy, S (Eq. (17)), is smoothly up-regulated in both prokaryotes and eukaryotes (Fig. 3(d) and (j), SI Figs. S5-S10(d) and S12-S22(d)), reflecting the general tendency that ζ becomes comparable to ξ on the IS (see bottom left region of Fig. 3(d) and (j)). Atomic environments on the IS and ES are thus subject to qualitatively different entropic constraints, likely also experiencing asymmetric perturbation responses. To sustain such a delicate metastable equilibrium, even instantaneously, requires the contribution of external forces, since otherwise the structural integrity of the IS could be compromised. The observed nonextensivity, where the entropy is largely indifferent to changes in the radial molecular size while being governed by the interaction range ratio ν (Eq. (2), Fig. 3(f) and (l), and SI Figs. S5-S10(f) and S12-S22(f)), is likely the key property ensuring that the structure can quickly react to environmental changes and visit unconventional metastable states. In summary, ν can be intuitively understood as a regulator of the NaVCh’s entropy-driven behavior, modulating the tendency of the components to either pack tightly or unpack.

According to (17), (20), we expect S to maximize in response to attenuation of repulsive effects (ζ→0). Consistent with this expectation, we find that the maximum of S typically occurs in the mid-pore region, where the equivalent of a hydrophobic core exists, namely, a prevalently hydrophobic central cavity (CC) (e.g., see Fig. 1 in [43]). In this mid-pore region, resisting structural disorder caused by excessive hydrophobicity-induced configurational freedom appears to be crucial: we observe that the PD tends to reduce its characteristic size, thereby decreasing its intrinsic dimension (SI S2.2) and, in turn, ensuring more efficient spatial organization.

#### 3.1.3 The PD/VSD structural transition as an order/disorder phase transition

Implications for the NaVCh functional architecture arising from the cancellation of interfacial tensions in the vicinity of the PD/VSD interface are best summarized by the membrane-perpendicular first-order hydropathic dipole field (HDF) amplitude, $h_{1,⊥}$ [kcal×Å] (as explained in SI 1.5.2).

The sign of $h_{1,⊥}{|}_{l_{i}}$ indicates whether the $h_{1,⊥}{\hat{e}}_{⊥}$-field induced by the PD at the current pore point is oriented extracellularly (↑) or intracellularly (↓) (Fig. 3 caption and SI 2.4.2, 2.5.2).

As shown in Fig. 3(e) and (k) and SI Figs. S5-S10(e), S12-S22(e), distinguishing between $h_{1,⊥,↑}$ and $h_{1,⊥,↓}$ highlights the critical [30], [31] nature of the inflection point.

Specifically, $h_{1,⊥,↓}$ and $h_{1,⊥,↑}$ exhibit clear negative and positive peaks, respectively, for $l\approx l_{i}$, indicating that $\left|h_{1,⊥}\right|$ is globally maximized. This, in turn, implies that the rate of change of $h_{1,⊥}$ has stagnated, causing the interfacial free-energy pair $\left\{\partial h_{1,⊥,↑}/\partial l,\partial h_{1,⊥,↓}/\partial l\right\}$ [kcal] to nearly vanish. This behavior is akin to a smooth (i.e., second-order) phase transition, in which, near the inflection point of the order parameter n, the associated response functions $h_{1,⊥,↓}$ and $h_{1,⊥,↑}$ exhibit sharp critical behavior.

Regardless of its orientation, $\left|h_{1,⊥}\right|$ increments in a power-law fashion in both prokaryotic and eukaryotic PDs. The corresponding scaling exponents, $\eta_{1,⊥,<}$ (Eq. (23)), are narrowly distributed with 3.2±0.6 and 3.5±1.3 (see Fig. 4(e) and (f), respectively). High Pearson correlation coefficients verify that these observations reflect a genuine self-similar increment behavior (e.g., see Fig. 3(c) from [34]). Microscopically, this necessitates that hydropathic dipoles connecting radial atom neighbors are nonrandomly aligned [34], since random alignment would make the self-similar incrementation of their cumulative index an extraordinary evolutionary accident.

The narrowness of the $\eta_{1,⊥,<}$-distribution (Fig. 4(e) and (f)), further necessitates a highly specialized nature for the water-mediated interactions established between the PD and the ion/water pore mixture. The PD operates under a narrow-banded dehydration protocol, through which ion selectivity can naturally emerge, as the radial alignment of dipoles around a pore point guarantees that water reorganization energies optimally dissipate into the atomic structure. Perturbation amplification is strictly unidirectional: perturbation amplitude attenuates inwards (toward the pore-lining interface, $\partial B_{0}$), while it amplifies outwards (toward the PD/VSD interface), irrespective of the current pore point. This implies the existence of a directed (or ordered) allosteric network inside the PD, where any pair of perturbed pore-lining constituents can potentially influence the same distant neighbor, unlike the other way around.

Once the characteristic PD size is surpassed (i.e., for $l>l_{i}$), $h_{1,⊥,↓}$ bends upwards, while $h_{1,⊥,↑}$ bends downwards, thereafter exhibiting transient behaviors that cannot be described by a single scaling rule, as inferred from the broad, zero-centered distribution of $\eta_{1,⊥,>}$ (Fig. 4(e) and (f)). $\left|h_{1,⊥}\right|$ may thus increment, decrement, or stagnate beyond the PD (note how percentile clouds broaden for $l>l_{i}$ in Fig. 3(e) and (k) and SI Figs. S5-S10(e), S12-S22(e)). Given the high Pearson correlation coefficients associated with distributional $\eta_{1,⊥,>}$-patterns on both sides of zero (Fig. 4(e) and (f)), the observed diversification of the scaling behavior of $\left|h_{1,⊥}\right|$ beyond the PD, supports that allosteric pathways coupling the PD to the VSDs are bidirectional: perturbations can be either attenuated or amplified from a VSD toward the PD/VSD interface in a pore-point-specific manner (SI 2.4.3, 2.5.3). This implies the multidirected (or disordered) nature of the allosteric network coupling the PD with the VSDs.

**Fig. 5:**
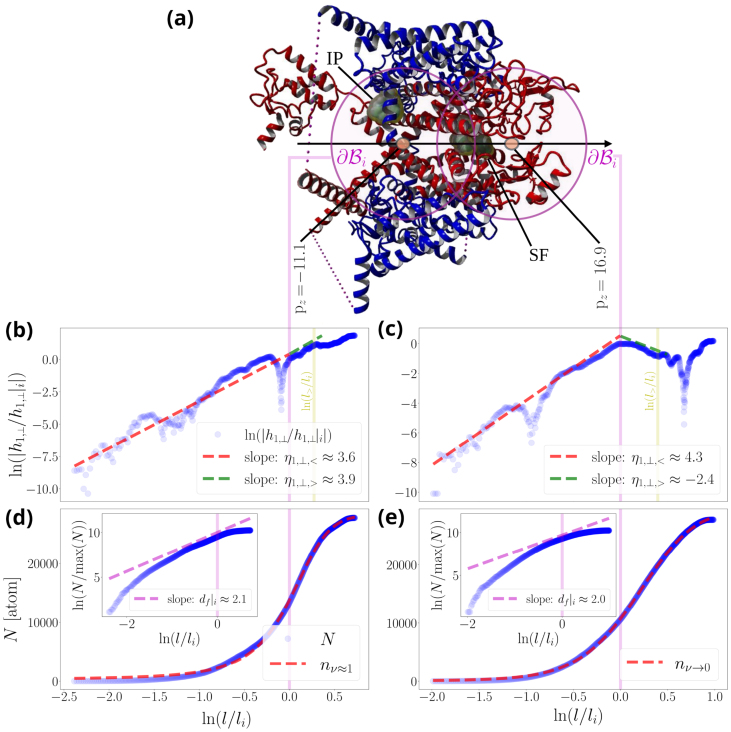
**Scaling of hydropathic dipole field around the NaV1.7 pore**. **(a)** Side view of a human NaV1.7 channel (PDB code: 7w9k). The pore domain (PD) and voltage sensor domains (VSDs) are illustrated in red and blue, respectively; the β subunits are omitted for clarity, and the van der Waals surfaces of the selectivity filter (SF) sequence Asp384/Glu942/Lys1422/Ala1714 and of the inactivation particle (IP) Ile1742/Phe1743/Met1744 are highlighted. Magenta circles represent the spherical surfaces $\partial B_{i}$ that delimit the characteristic size of the PD. **(b) and (c)** Example traces of the empirical cumulative atom number N(Eq. (32)) and its best-fit model n(Eq. (3)) on different sides of the pore, for representative pore points ${\text{p}}_{z}=-11.1$ (intracellular side) and ${\text{p}}_{z}=16.9$ (extracellular side). Insets show the intrinsic PD dimension $d_{f}{|}_{l_{i}}$(Eq. (19)). **(d) and (e)** Example traces of the empirical membrane-perpendicular hydropathic dipole field $h_{1,⊥}$(Eq. (21)) acting on different sides of the pore. The power-law exponents are computed over the radial extent of the PD and the VSDs according to the general rule outlined in Eq. (23). Associated Pearson correlation coefficients quantify the goodness of the power-law approximation and are >0.9 for all cases. Molecular illustrations were generated using Yasara software [92].

To exemplify, we consider the NaV1.7 molecule and illustrate the scaling of N (and n) alongside $\left|h_{1,⊥}\right|$ on the two sides of the pore: (i) the ES side of the Asp384/Glu942/Lys1422/Ala1714 SF sequence, where an ion-attracting funnel is formed, and (ii) the AG side, where the inactivation particle (Ile1742/Phe1743/Met1744 motif) is located (Fig. 5(a)). N and n show excellent agreement, demonstrating the importance of correctly estimating the value of ν along the pore (Fig. 5(b) and (c)). On the ES side, ν→0 indicates that the atomic environment is packed so as to effectively attenuate repulsive interactions, whereas at the AG, ν→1 indicates that repulsive interactions have recovered and become comparable to the attractive interaction range (Fig. 5(c) vs. (b)). This difference in atom packing appears to leave the intrinsic dimension of the PD largely unaffected, showcasing how the PD can readjust its characteristic size under substantial ν-variations to preserve structural and functional integrity as the range of repulsive interactions changes (insets in Fig. 5(b) and (c), together with Eq. (19) and Section 3.1.1). The scaling of $\left|h_{1,⊥}\right|$ exhibits a similar trend in both cases along the characteristic radial extent of the PD, with the corresponding scaling exponents lying close to one another and assigned high PC values, in agreement with our expectations from Fig. 4(f) (Fig. 5(d) and (e)). The key difference arises beyond the characteristic PD size, where $\left|h_{1,⊥}\right|$ bends downward on the ES side but continues to increase on the IS side, approximately obeying the same power law, revealing that perturbations on the two sides of the NaV1.7 pore are differentially processed (Fig. 5(d) vs. (e)). Implications of this observation for NaV1.7 mutational robustness in the context of human pain disease are examined in detail below.

### 3.2 NaV1.7 mutational robustness in the context of human pain diseases

Let us assume that the NaV1.7 molecule exhibits a uniform mutational robustness profile, meaning the molecule’s ability to tolerate mutations is approximately the same across all of its constituent parts. Given that the PD/VSD interface is the most occupied molecular surface, it then follows that this region is also a mutation hotspot, i.e., a geometric site where the likelihood of residue mutation is highest.

To scrutinize the validity of this assumption, we examine mutation clustering in the highest-resolution NaV1.7 structure currently available (PDB code: 7w9k). We consider all *SCN9A*-gene-related mutations found in gnomAD [23] and ClinVar [64] databases, yielding $N_{\text{mut}}=1705$ events spanning three main categories: pathogenic (6.5 %), non-pathogenic (11.3 %), and variants of uncertain significance (VUS) (82.2 %). The set of pathogenic mutations contains the subset of pain-disease-associated mutations (2.7 %) and a substantial portion of pain-disease-unrelated pathogenic mutations (3.8 %). Pain-disease-associated mutations are categorized as either gain-of-function (GoF) (2.1 %) or loss-of-function (LoF) (0.5 %) (SI S1.9). Non-pathogenic mutations comprise two subsets: benign and neutral, with the latter consisting of carefully selected negative controls for human pain disease, sourced from Refs. [43], [65].

To probe mutation clustering relative to the PD/VSD interface, we consider the dataset $\left\{l_{\text{mut}}-l_{i}\right\}$, where, for each of the $N_{\text{mut}}$ mutations, $l_{\text{mut}}$ is the Euclidean distance between a pore point and the geometric center of the residue undergoing mutation (its ‘structural location’). The following rule determines whether the mutational robustness uniformity assumption is accepted or rejected: if, at a given pore region, the mean, mode, and median of $l_{\text{mut}}-l_{i}$ vanish simultaneously, then the likelihood of observing a mutation is maximized at $l=l_{i}$, and the underlying mutation distribution is approximately bell-shaped, assigning roughly equal mutation weight (in a statistical sense) to the PD and the VSDs.

**Fig. 6:**
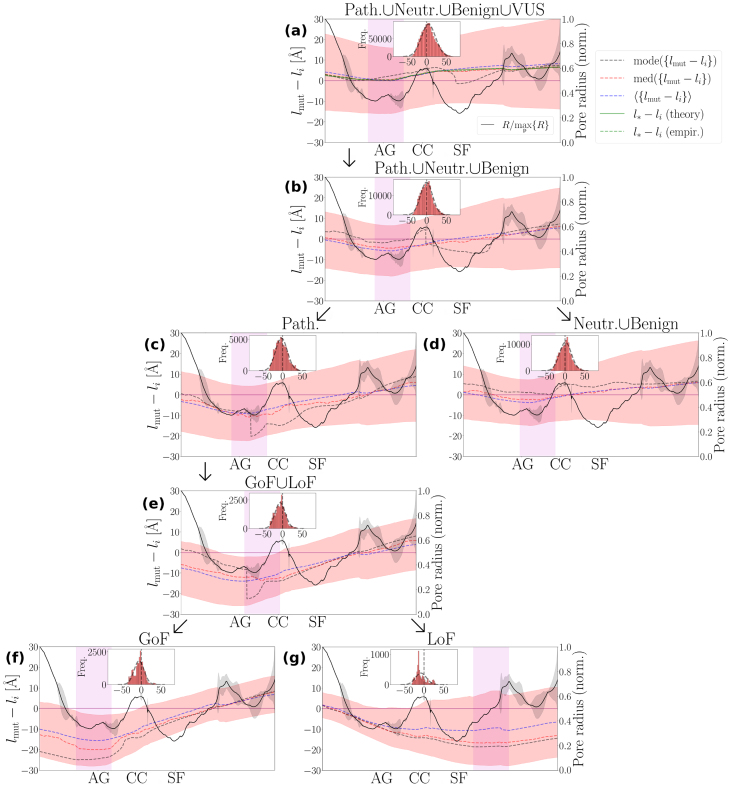
**Distributional characteristics of mutations across the NaV1.7 structure**. We summarize the statistical properties of the $\left\{l_{\text{mut}}-l_{i}\right\}$ dataset in terms of the median, med(⋅), and the mean, ⟨⋅⟩, for pore points p∈P. Here, $l_{\text{mut}}$ denotes the Euclidean distance between p and the geometric center of the residue being mutated. We break down $\left\{l_{\text{mut}}-l_{i}\right\}$ into subsets, each corresponding to a different mutation set. Insets visualize the histogram of the $\left\{l_{\text{mut}}-l_{i}\right\}$ dataset, i.e., the collapsed distribution incorporating contributions from all pore points. In **(a)**, we show the $\left\{l_{\text{mut}}-l_{i}\right\}$-distribution of the parent set of all (i.e., Path.∪Benign∪Neutr.∪VUS) *SCN9A*-gene mutations. $l_{*}$ identifies the structural location where the coin-flipping entropy is maximized. Note that if $n_{L}\approx K$, $l_{*}$ can be approximated by $l_{i,\text{b}}$(Eq. (8)). The empirical and theoretical instances of $l_{*}$ are determined by the l values for which $N/N_{\text{total}}=0.5$ and $n/n_{L}=0.5$ are satisfied, respectively. The parent dataset is broken down as follows: **(b)**, pathogenic and non-pathogenic (Path.∪Benign∪Neutr.). **(c)**, pathogenic (Path). **(d)**, non-pathogenic (Benign∪Neutr.). **(e)**, gain-of-function (GoF) and loss-of-function (LoF) (GoF∪LoF). **(f)**, GoF. **(g)**, LoF. Note that a negative and positive value of $l_{\text{mut}}-l_{i}$ indicates that the residue being mutated is likely residing in the PD and the VSDs, respectively. The areas highlighted in light magenta represent the pore regions where the median is minimized.

We report that the uniform mutational robustness hypothesis for the NaV1.7 molecule is rejected along P, except at the AG pore region (Fig. 6(a)), as explained below.

While the mean and median of $\left\{l_{\text{mut}}-l_{i}\right\}$ closely agree, and the mode oscillates around them within reasonable bounds (Fig. 6(a)), their systematic deviation from zero reflects random genetic fluctuations governed by a flipping-coin maximum entropy principle. Accordingly, the probabilities of a mutation occurring inside or outside B are given by $P_{\text{in}}=N/N_{\text{total}}\approx n/n_{L}$ and $P_{\text{out}}=1-P_{\text{in}}$, respectively. Maximizing the flipping-coin entropy, $-P_{\text{in}}\log_{2}(P_{\text{in}})-P_{\text{out}}\log_{2}(P_{\text{out}})$, requires that $P_{\text{in}}=P_{\text{out}}=n_{*}/n_{L}=0.5$, with $l_{*}$ denoting the l-value for which $n_{*}/n_{L}=0.5$ is satisfied.

**Fig. 7:**
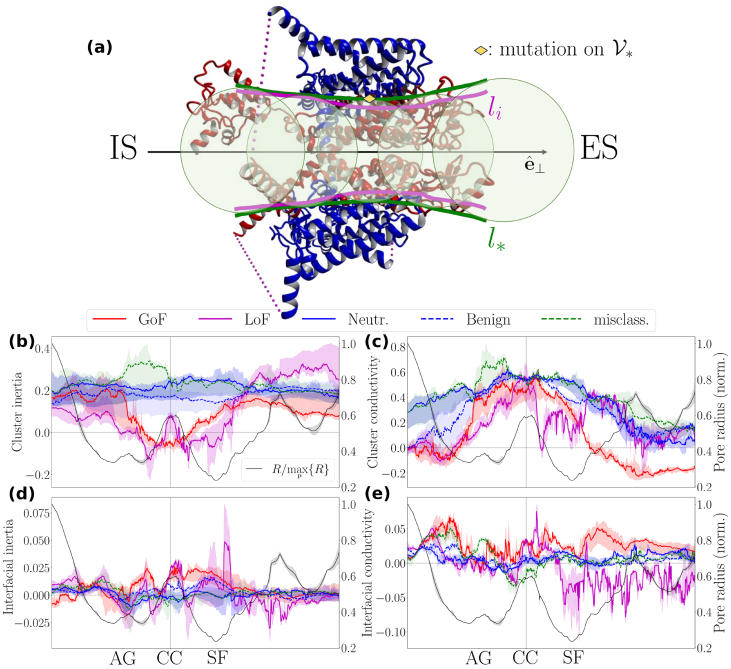
**Inertia and conductivity profile of structural locations attracting pain-disease-associated mutations**. **(a)**, Side view of a human NaV1.7 channel (PDB code: 7w9k). The PD and VSDs are illustrated in red and blue, respectively. For clarity, the β subunits are not shown. The dense arrangement of $B_{*}$-balls along P creates the smooth cylindrical surface (in green), denoted $V_{*}=\partial \underset{p}{⋃}B_{*}$. For comparison, the characteristic size of the PD, $l_{i}$, is also illustrated (in magenta). Note that the inequality $l_{*}>l_{i}$ arises from the atom-packing condition ν<1 (SI Fig. S23). At the AG pore region, where ν↗1 (q↗2), $l_{*}\approx l_{i}$ implies $l_{*}\approx l_{i,\text{b}}$ with $n_{L}\approx K$ (see Eq. (8)). In **(b)** and **(c)**, we statistically summarize the n-cluster inertias, $\phi_{2k}$, and the n-cluster conductivities, $\phi_{2k+1,⊥}$, characterizing structural locations where gain-of-function (GoF), loss-of-function (LoF), neutral (Neutr.), and benign (Benign) mutations appear, for k=0,1,…,5. **(d)** and **(e)** provide analogous information at the interface level: we statistically summarize the ∂n-shell, inertias $I_{2k}$, and the ∂n-shell conductivities, $I_{2k+1,⊥}$. Clouds around the index traces represent the min–max range of the underlying data points. The misclassified (misclass.) subset contains pain-disease-associated mutations that are systematically misclassified by the machine-learning algorithm (see Fig. 8(b). The conductivity index derived from the first-order hydropathic dipole field is shown separately in SI Fig. S4, as the pair $\left\{h_{1,+,⊥},h_{1,-,⊥}\right\}$ satisfies Eq. (24) only partially (SI S2.3.1). The construction of the statistical summary indices of $\phi_{j}$ and $I_{j}$ is described in SI S1.8.2. Molecular illustrations were generated using Yasara [92].

As shown in Fig. 6(a), the line $l_{*}-l_{i}$ coincides with the $\left\{l_{\text{mut}}-l_{i}\right\}$-median, verifying that mutation clustering in the NaV1.7 is predominantly shaped by the flipping-coin randomness. Across an evolutionary time-scale, this results in a bell-shaped mutation distribution approximately centered at $l=l_{*}\overset{n_{L}\approx K}{\approx }l_{i,\text{b}}$ (Eq. (8)), ensuring that molecular diversity is explored inside and outside $B_{*}$ in an unbiased manner. Since the range of attractive interactions prevails over the range of repulsive interactions (SI Fig. S23), the $\partial B_{*}$-interface covers the PD/VSD interface (Fig. 7(a), thus serving as a buffer zone that absorbs mutation-induced perturbations and mitigates their impact on the PD.

In contrast, this is not the case at the AG pore region, where all statistical indices considered above tend to vanish (Fig. 6(a)), consistent with a uniform mutational robustness profile as $\partial B_{*}$ coincides with the PD/VSD interface (Fig. 7(a)). Although the PD loses its protective buffer, it gains evolutionary flexibility, allowing for greater exploration of molecular diversity. Moreover, mutations around the AG pore region weigh equally on the PD and the VSDs, preventing either from being disproportionately stressed.

Collapsing $\left\{l_{\text{mut}}-l_{i}\right\}$ into a distribution yields a roughly symmetric bell shape, well described by a normal distribution with mean of 4.46 and standard deviation of 18.04 (inset in Fig. 6(a)). Excluding VUS shifts the distribution to the left, to −0.47±15.18 (Fig. 6(b)). Pathogenic and non-pathogenic mutation distributions have means and standard deviations of −2.89±14.9 and 0.9±15.1, respectively, indicating a slight preference for the PD and the VSDs (Fig. 6(c) and (d)). The pain-disease-associated mutation distribution is further left-shifted, with −6.2±13.0 (Fig. 6(e)), indicating a strong preference for the PD.

Distinguishing between GoF and LoF mutations reveals opposite statistical trends (Fig. 6(f) and (g)). GoF mutations are concentrated near the AG (Fig. 6(f)), whereas LoF mutations converge toward the ES of the SF, where the extracellular funnel is formed (Fig. 6(g)). The molecular basis for GoF and LoF pain phenotypes can thus be rationalized in terms of qualitatively different perturbation modes. Positive $\eta_{1,⊥,>}$ exponents at the AG pore region favor GoF-triggered perturbations to amplify and propagate further into the VSDs (SI Fig. S23). By contrast, negative $\eta_{1,⊥,>}$ exponents on the ES side of the pore disfavor further amplification of LoF-triggered perturbations into the VSDs (SI Fig. S23), potentially causing them to be locally absorbed, with detrimental impact on the PD.

#### 3.2.1 Violation of inertia and conductivity constraints underpins human pain disease at the molecular level

Let us further assume that pain-disease-associated mutations exploit imbalances in the interaction network at the interface level to perturb the underlying n-cluster.

To scrutinize this assumption for NaV1.7, we utilize the Decomposition ansatz (24) (SI S2.3) to derive the logarithmic composite susceptibility $\phi_{j}$ (Eq. (29)) and combine it with the interfacial coupling strength $I_{j}$ (Eq. (28)) in the form of the ratio $I_{j}/\phi_{j}$ (with $\phi_{j}\neq 0$). The quantity $I_{j}/\phi_{j}$ establishes an upper bound on the number of available perturbation modes encoded in a structural location (SI Eq. (S24)). Simply put, $I_{j}/\phi_{j}$ measures how many ways there are to destabilize an n-cluster, thus serving as a perturbation potential (i.e., as a measure of the sandpile slope; SI Eq. (S24)) associated with the structural location under scrutiny.

A mechanical analogy rationalizing $I_{j}/\phi_{j}$ is that of a maximum torque/force principle. When tightening a bolt, applying force at a stable far-end grip, where the inertia is minimal, maximizes torque. Analogously, residues distributed over strongly coupled interfaces covering n-clusters of vanishingly small inertia (probed with $\left|\phi_{2k}\right|$, k=0,1,…,5) are ideal mutagenesis sites, as they can effortlessly perturb the n-cluster rotational profile. A similar mechanism governs perturbations of the n-cluster conductivity profile, characterized by the transverse odd-parity moments $\left|\phi_{2k+1,⊥}\right|$ (k=0,1,…,5). A vanishingly small HDF amplitude indicates that the n-cluster conductivity becomes highly susceptible to perturbations, such that even minor surface fluctuations can induce disproportionately large reorganizations in both the amplitude and orientation of the local dehydration forces.

In contrast to non-pathogenic mutations, pain-disease-associated mutations prefer interfaces covering n-clusters whose inertia decreases when approaching the CC pore region from both sides and, additionally, at the SF and AG pore regions (Fig. 7(b)). Specifically, the inertia of the n-clusters whose surfaces act as hotspots for GoF and LoF mutations vanishes twice and four times, respectively, at approximately symmetric locations along P. The arrangement and number of these inertia zero-crossings may detrimentally perturb the NaV1.7 functional architecture by introducing excess rotational atom-packing degrees of freedom (DoF). Whether this leads to an increased or decreased pore-open probability and, consequently, to a GoF- or LoF-like electrophysiological signature is likely set by a threshold in the number of inertia zero-crossings, which determines the tolerable excess rotational atom-packing DoF before the gating cycle collapses. Targeting binding sites in the CC with anchoring molecules (e.g., local anesthetics) can therefore offer a rational strategy for restoring inertia and mitigating the effects of the perturbation.

Additionally, GoF and LoF mutations prefer interfaces covering n-clusters whose conductivity is diminished beyond the AG pore region toward the IS (Fig. 7(c)). GoF mutations induce a conductivity zero-crossing on the ES of the SF pore region, whereas LoF mutations suppress conductivity on both sides of the SF without producing a definitive sign change exactly at the SF: the LoF-related trace in Fig. 7(c) approaches zero and fluctuates around it between the SF and the CC, as well as on the ES side of the SF. Pain-disease-associated mutations thus appear to maximally perturb HDFs in the vicinity of the SF and AG, but not exactly at them, thereby potentially altering ion/water fluxes precisely at mediator pore regions where the pore radius is rapidly changing.

At the interface level, inertia and conductivity amplitudes associated with pain-disease-related mutations fluctuate more strongly than those associated with non-pathogenic mutations (Fig. 7(d) and (e)). This suggests that GoF and LoF surface hotspots engage in substantially stronger coupling interactions with their environment compared to surfaces attracting non-pathogenic mutations. Moreover, GoF and LoF surface hotspots are characterized by oppositely signed interfacial conductivities at the ES of the CC, implying that, once perturbed, these interfaces could generate oppositely directed ion/water flux responses (Fig. 7(e)).

#### 3.2.2 Verification via machine learning experimentation

We adopt a simple and transparent machine learning strategy based on a stratified k-fold cross-validated support vector machine classifier with a radial basis function kernel, applied locally (per pore point) and then globally on features summarized via median statistics (for details, see SI S1.10). This two-stage setup is reminiscent of meta-learning, as the global model builds on information extracted by the local models, yet we intentionally avoid hyperparameter tuning or complex architectures so that $I_{j}/\phi_{j}$-derived features – and their permutation-based importances – remain central to the analysis. To avoid divergent $I_{j}/\phi_{j}$ values, we treat $\phi_{j}$ and $I_{j}$ as two separate feature inputs and also investigate the significance of inertia- and conductivity-related constraints separately, ending up with four feature inputs: $\left\{\phi_{2k},I_{2k},\phi_{2k+1,⊥},I_{2k+1,⊥}\right\}$.

##### 3.2.2.1 Machine learning experiment I: pain-disease-associated vs. neutrals (PDB: 7w9k)

Following previous works [35], [65], we apply our algorithm to a well-balanced dataset comprising pain-disease-associated mutations and neutral variants. The local performance of the classifier, evaluated in terms of the area under the curve (AUC) and F1 scores, remains generally stable along P, indicating that the classifier can adapt well to local atom-packing conditions (SI S2.6.1).

**Fig. 8:**
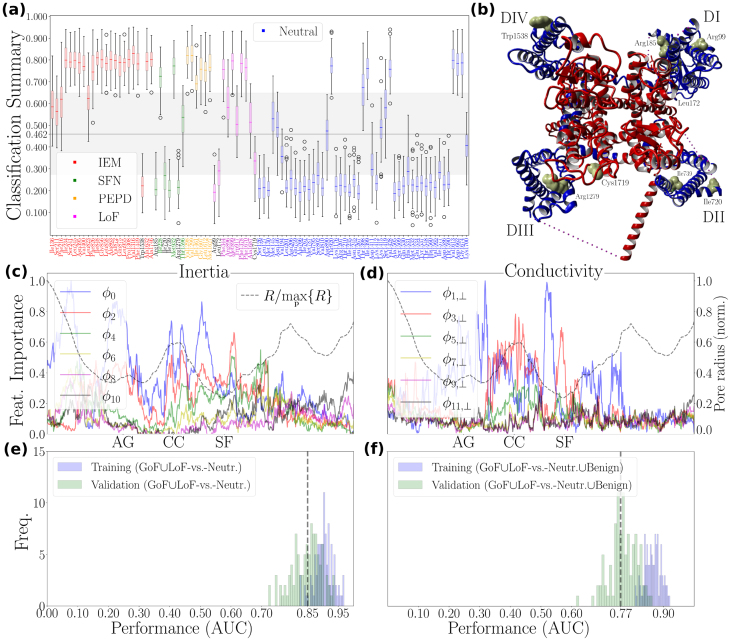
**Machine-learning-assisted verification: pain-disease-associated mutation hotspots exhibit substantially distinct perturbation potential profile**. **(a)**, Summary of a machine-learning experiment evaluating the distinctiveness of the pain-disease-associated class (class_0: GoF∪LoF) relative to the neutral class (class_1: Neutr.). The gain-of-function (GoF) subclass comprises structural locations whose mutability is phenotypically associated with inherited erythromelalgia (IEM), small fiber neuropathy (SFN), and paroxysmal extreme pain disorder (PEPD) (SI Tab. S3). The loss-of-function (LoF) subclass comprises structural locations whose mutability is phenotypically associated with insensitivity to pain (SI Tab. S3). Neutrals are structural locations whose mutability is not likely to be phenotypically associated with human pain disease, sourced from [35], [65]. The optimal threshold at 0.462 corresponds to the median of the best thresholds obtained through bootstrapping; each bootstrap sample of class_0 probabilities yields a threshold that maximizes the F1-score. X-axis ticks indicate the structural location of a mutation event in the NaV1.7 structure. X-axis ticks of misclassified pain-disease-associated mutations (i.e., those that do not pass the linear threshold) are highlighted in black. **(b)**, Top-view illustration of the human NaV1.7 channel (PDB code: 7w9k). For clarity, the β-subunits are not shown. The van der Waals surface of misclassified structural locations is highlighted in yellow. The PD and the VSDs are shown in red and blue, respectively. In **(c)** and **(d)**, we plot the permutation-based importance scores for the features $\phi_{2k}$ and $\phi_{2k+1,⊥}$, k=0,1,…,5, which account for inertia and conductivity constraints at the cluster level, respectively, obtained via 200 permutation rounds per pore point (per feature set) (see SI S1.10 for details). In **(e)** and **(f)**, we illustrate the distribution of the final area-under-the-curve (AUC) scores obtained from the final classification round for two different machine-learning experiments, namely, the one summarized in (a) and another with class_0: GoF∪LoF vs. class_1: Neutr.∪Benign. Benign denotes structural locations whose mutability is not likely to be associated with disease (SI S1.9). Details concerning the machine-learning algorithm design and parameter selection can be found in SI S1.10. Molecular illustrations were generated using Yasara [92].

A yes/no variant classification scheme based on a linear threshold is illustrated in Fig. 8(a). Its performance, evaluated in terms of the median AUC, reaches 0.85 (Fig. 8(e) and Table 1).

**Table 1:** Machine learning experiments summary. Median area-under-the-curve (AUC) and F1 scores obtained during the final classification round (SI 1.10.2). The first and second numbers of each (⋅,⋅)-pair are median values obtained during training and testing the final classification model, respectively. The thermostability significance of inertia (iner.) and conductivity (cond.) constraints is probed by the feature inputs $\left\{\phi_{2k},I_{2k}\right\}$ and $\left\{\phi_{2k+1,⊥},I_{2k+1,⊥}\right\}$, respectively. *SCN9A*-gene mutation datasets I, II, and III contain the classes {class_0: GoF∪LoF, class_1: Neutr.}, {class_0: GoF∪LoF, class_1: Neutr.∪Benign}, and {class_0: Path., class_1: Neutr.∪Benign}, respectively. GoF and LoF stand for gain-of-function and loss-of-function, respectively, and represent mutations associated with increased or diminished pain sensation (listed in SI Tab. S3). Neutrals (neutr.) are carefully selected human pain disease negative controls, sourced from [35], [65]. Benign variants are generally not expected to be associated with a disease phenotype. Note that the pathogenic (path.) class contains both pain-disease-associated and pain-disease-unrelated pathogenic mutations.

	PDB: 7w9k, res.: 2.2 Å			PDB: 6j8j, res.: 3.2 Å		
Constraint	iner./cond.	iner.	cond.	iner./cond.	iner.	cond.
AUC (dataset I)	(0.90,0.85)	(0.89,0.83)	(0.87,0.84)	(0.90,0.83)	(0.87,0.82)	(0.87,0.83)
F1 (dataset I)	(0.81,0.78)	(0.78,0.76)	(0.81,0.80)	(0.80,0.78)	(0.79,0.76)	(0.80,0.78)
AUC (dataset II)	(0.87,0.77)	(0.80,0.74)	(0.85,0.77)	(0.84,0.72)	(0.79,0.70)	(0.79,0.72)
F1 (dataset II)	(0.75,0.72)	(0.76,0.74)	(0.74,0.72)	(0.79,0.73)	(0.80,0.78)	(0.74,0.70)
AUC (dataset III)	(0.77,0.63)	(0.71,0.60)	(0.71,0.64)	(0.79,0.65)	(0.74,0.64)	(0.71,0.61)
F1 (dataset III)	(0.71,0.62)	(0.67,0.61)	(0.68,0.64)	(0.72,0.62)	(0.68,0.61)	(0.67,0.59)

The $\phi_{2k}$ feature-importance profile shows that maintaining higher-order interactions becomes increasingly important around the SF pore region. The importance of the second- and fourth-order inertia features exceeds that of the zeroth-order feature, suggesting that rotational and vibrational modes up to at least fourth order may influence ion selectivity (Fig. 8(c)). Corroboratively, the importance of higher-order interfacial inertia features rises sharply at the SF pore region, while, strikingly, the zeroth-order term $I_{0}$ loses significance (SI Fig. S26(a)).

Additionally, at the SF pore region, higher-order conductivity features – particularly those of third and fifth order – become increasingly dominant, even surpassing the importance of first-order features (Fig. 8(d)). This indicates that volumetric, potentially asymmetric interactions may be crucial for the dehydration of sodium ions. In the $I_{2k+1,⊥}$ feature domain, we observe similar dependencies at the ES entry point of the SF (SI Fig. S26(b)).

To clarify whether these results depend on the sign of the features, which encode information about the direction of the perturbation response ((22), (27)), we repeat the machine learning experiment with the unsigned feature input $\left\{|\phi_{2k}|,|I_{2k}|,|\phi_{2k+1,⊥}|,|I_{2k+1,⊥}|\right\}$. Classification performance becomes only marginally worse (Table 1 vs. SI Tab. S5), suggesting that the NaV1.7 functional architecture is affected by violations of the inertia and conductivity constraints shown in Fig. 7 in a largely direction-independent manner.

##### 3.2.2.2 Machine learning experiment II: pain-disease-associated vs. non-pathogenic (PDB: 7w9k)

To demonstrate that our findings are not biased by the specific choice of neutrals, we repeat the machine learning experiment, this time focusing on the non-pathogenic mutation subset containing both neutral and benign mutations.

The resulting AUC scores have a median of 0.77 (Fig. 8(f) and Table 1). Despite considerable class imbalance – non-pathogenic mutations greatly outnumber pain-disease-associated ones, with a class ratio as low as 0.19 – the algorithm maintains both accuracy and stability, showing no detrimental effects (SI Figs. S24(c), S25(c)). These findings reinforce that pain-disease-associated mutations occur in molecular neighborhoods with a perturbation-potential profile that differs substantially from that of non-pathogenic mutations.

##### 3.2.2.3 Machine learning experiment III: pathogenic vs. non-pathogenic (PDB: 7w9k)

Running our algorithm on the entire *SCN9A*-gene mutation dataset results in a performance drop of approximately 25%, with the median AUC score decreasing to 0.63 (Table 1). This reduction is primarily driven by the inclusion of pain-disease-unrelated pathogenic mutations. The perturbation-potential profile of structural locations whose mutability is associated with disease phenotypes distinct from painful or painless neuropathies resembles that of non-pathogenic mutations, at least up to the first derivative of $\phi_{j}$. Our confidence in this finding is high, as fewer than 6% of all pain-disease-unrelated pathogenic mutations are characterized as ‘Pathogenic/Likely pathogenic’ or ‘Likely pathogenic’.

Enhancing classifier performance would likely require incorporating higher-order derivatives of $\phi_{j}$ together with more refined scaling-exponent estimates, although noise becomes progressively harder to control with increasing derivative order and distance from the pore. Even with the current implementation, however, the gains from adding $I_{j}$ are very small, if present at all, which is plausibly explained by intrinsic structural noise and the noise sensitivity of derivative-based quantities (SI Tab. S5). We nevertheless report results for the combined feature set in the main text, to avoid relying solely on $\phi_{j}$ and to verify that including its derivative-based counterparts $I_{j}$ does not alter the conclusions, thereby reducing the risk of bias toward a single representation of the thermostability constraints. In line with these limitations, pain-disease-associated mutations affecting the VSDs are more likely to be misclassified (Fig. 8(b)). Four out of five misclassified GoF mutations are associated with an SFN phenotype and occur at VSD locations Arg185 (DI), Ile720 (DII), Ile739 (DII), and Arg1279 (DIII), while the fifth, linked to an IEM phenotype, is located at Trp1538 (DIV). Among the three misclassified LoF mutations, Arg99 (DI) and Leu172 (DI) reside in the VSD, and Cys1719 (DIV) lies in the extracellular loop connecting S5 and S6.

##### 3.2.2.4 Repeating machine learning experiments I–III using a lower-resolution structure (PDB: 6j8j)

To assess how lower resolution impacts algorithm performance, we repeat the analysis using a widely studied NaV1.7 structure with lower resolution (PDB code: 6j8j). Despite a slight drop in performance, the results remain largely consistent (Table 1 vs. SI Tab. S5). Given that both the high- and low-resolution structures likely represent an inactivated state of the channel, the observed decrease in performance can be attributed to the limitations imposed by lower structural resolution.

##### 3.2.2.5 Inertia-vs-conductivity: what matters more for NaV1.7 physiological functioning? (PDB: 7w9k, 6j8j)

Exchanging the full feature set $\left\{\phi_{2k},I_{2k},\phi_{2k+1,⊥},I_{2k+1,⊥}\right\}$ with either $\left\{\phi_{2k},I_{2k}\right\}$ or $\left\{\phi_{2k+1,⊥},I_{2k+1,⊥}\right\}$ for the 7w9k or 6j8j structures results in a drop in algorithm performance that is always less than 10 % (Table 1, SI Tab. S5). Additionally, the AUC scores achieved with $\left\{\phi_{2k},I_{2k}\right\}$ are close to those obtained with $\left\{\phi_{2k+1,⊥},I_{2k+1,⊥}\right\}$. Together, these results suggest that the feature sets $\left\{\phi_{2k},I_{2k}\right\}$ and $\left\{\phi_{2k+1,⊥},I_{2k+1,⊥}\right\}$ contain redundant information, implying that rotational and translational NaV1.7 degrees of freedom (DoF) are coupled. The corresponding perturbation modes are thus likely intertwined through feedback, with molecular rotations around the pore influencing the flow of ions through the pore, and vice versa.

## 4 Discussion

The NaVCh functional architecture represents the latest product of an evolutionary process initiated nearly three billion years ago [66]. It embodies a rich repertoire of metastable dynamics emerging from the instantaneous coordination of thousands of atoms assembled into several hundred amino acids and structurally organized into distinct domains around a central axis. Parsimonious models of the NaVCh functional architecture must therefore rely on evolutionarily conserved laws that connect the microstructure (single atom) with the macrostructure (multi-domain molecule) in a way that allows molecular functionality to emerge *a priori*, i.e., by virtue of the relationships established among these evolutionarily conserved laws. Since an atom is separated from a domain by orders of magnitude in length scale, our quest finds a natural place within the RG framework, which clarifies how (i.e., through which scaling operations) one can transition from one molecular scale to the next without losing the ability to reconstruct essential functional characteristics of the molecule. Because the evolutionarily conserved laws are precisely defined by the underlying scaling operations, the two concepts are interchangeable and are both encompassed by the term ‘scaling law.’ These considerations are summarized in the key analytical result of the RG flow equation (Eq. (30)), which is applied in a pore-point-specific manner, thereby establishing a connection between the porous microenvironment and the surrounding molecular macroembedding context.

Importantly, we arrived at Eq. (30) by starting from the simplest possible equation of state (Eq. (2)), which describes the number of atoms within an infinitely thin shell ∂B with a repulsive and a stabilizing term, yet is still capable of supporting a pore-forming macroenvironment in which a PD is radially succeeded by four VSDs. To identify the PD/VSD interface in an unbiased manner, we focused on NaVCh shape characteristics, specifically the sign change in the curvature of the ‘slow’ [67] state variable n, which marks a prominent inflection point. We are therefore confident that the logical thread connecting Eq. (2) with Eq. (30) is well-founded, suggesting that renormalizability is a fundamental property of NaVCh protein molecules. It is thus unsurprising that our theoretical framework can recapitulate Widom’s scaling law [68] under mean-field-like conditions, and does so at a molecular scale matching the inflection point (SI S1.7.4), indicating that NaVChs belong to the same universality class as other multi-domain complex systems with some degree of fluidity, whose constituents interact via magnetic-like fields [69].

Experimental support comes from the analysis of sufficiently resolved, all-atom NaVCh structures, which adhere to Eq. (2) within reasonable structural constraints – deviating only when the PD is extended with a C-terminal domain and the VSDs are removed (SI Fig. S7). The NaVCh structure can thus be treated as an ‘extended’ [70] (or ‘transient’ [71]) self-similar object whose intrinsic dimension changes continuously with molecular scale (Eq. (11)). A statistical-mechanical description of such ‘extended’ fractals is available within the framework of nonextensive statistical mechanics [51], [52], hallmarked by the q-generalized atom-packing entropy given by Eq. (17). Juxtaposing the notions of intrinsic dimension and atom-packing entropy suggests that formation of the central cavity is a structural consequence of hydrophobicities being buried inside the NaVCh core over evolutionary timescales (3.1.1 and SI S2.2). Accordingly, excess atom-packing degrees of freedom generated by increasing core hydrophobicity are compensated by a reduction in intrinsic dimension, thereby preserving the structural integrity of the PD sub-architecture, as described by Eq. (19). This phenomenon is generally interpreted within the evolution-driven dimensionality reduction framework [72], which favors more efficient spatial organization in eukaryotic NaVChs, as verified both here across the NaVCh superfamily and across thousands of proteins in Ref. [63]. The physical basis of this phenomenon lies in the long-range nature of water-mediated interactions, which allow spatially distant residues to influence one another [73], [74], [75]. Consistent with previous experimental findings reported in Refs. [58], [59], [60], [76], we find that the amplitude of the effective hydrophobic ‘force’ holding distant components together decays exponentially with increasing molecular scale, exhibiting a characteristic decay length, given by ξ, on the order of 10 Å (Fig. 4(c) and (d)).

Self-similarity implies long-range interactions, which in turn lead to synergistic constituent functioning via allostery [77]. The NaVCh field is well positioned to begin contemplating the role of such synergies within NaVCh molecules and their significance in both physiological function and mutation-perturbed, disease-related contexts (e.g., see [78], [79], respectively). Our results suggest that the *modus operandi* of the PD primarily relies on synergy built upon a narrow-width hydropathic dipole field (HDF) exponent distribution (Section 3.1.3). We argue that this evolutionary trend effectively reduces the number of interaction modes required to peel water molecules from a sodium ion while simultaneously increasing the number of available pathways through which dehydration free energy can dissipate rapidly and deeply – i.e., in a nearly adiabatic manner – into the PD sub-architecture. Synergies are thus expected to arise from the unidirectional (outward-radiating) nature of allosteric interactions within the PD, enabling collective responses to perturbations, as observed in molecular dynamics simulations [80]. Beyond the PD, the directionality of allosteric interactions becomes diversified, supporting the notion that VSD constituents may engage in asymmetric interactions both among themselves and with the PD. This renders analysis of VSD information-processing capabilities challenging, calling for more detailed future investigations that examine each VSD individually. Nevertheless, even with our current coarse-grained approach, we infer that eukaryotic VSDs possess more specialized information-processing capabilities than their prokaryotic counterparts, enabling differential perturbation processing on the ES and IS (SI S2.5.3).

Our understanding of the molecular basis of human pain disease has been built upon painstaking experimental efforts, primarily relying on cell-based electrophysiology assays that screen single-amino-acid mutations in the human NaV1.7 molecule (for a list, see SI Tab. S3). Resolving the structure–function relationship for human NaV1.7 in the context of human pain disease can seem like assembling an almost impossible puzzle: each piece encodes only a tiny fraction of the relevant biophysical and neurophysiological possibilities. To demonstrate how our theoretical framework can simplify this task, we mapped all *SCN9A* gene mutations onto a wild-type NaV1.7 molecule and attempted to explain the observed mutation clustering patterns (Section 3.2). We identified the most frequently mutated interface and showed that it becomes indistinguishable from the PD/VSD interface around the AG, where mean-field conditions are established as q↗2. This finding indicates that the NaV1.7 mutation landscape has not emerged purely by chance but is constrained by NaV1.7 shape features that vary along the pore and are effectively captured by q. A tendency toward a more globular-like shape lowers the risk of experiencing a detrimental perturbation: as the PD/VSD interface becomes the primary mutagenesis site, mutations tend to land between the PD and VSD, thereby sparing either domain from direct damage. Pain-disease-associated mutations appearing at the AG within the PD are most likely associated with a GoF phenotype, consistent with the mutation clustering patterns reported in Ref. [81]. This pattern is reversed for mutations linked to a LoF pain phenotype (Fig. 6, panel (g) vs. (f)); however, the limited number of available LoF pain mutations precludes any definitive conclusions. Nevertheless, when viewed through the RG lens, these observations suggest that the primary molecular distinction between GoF and LoF pain phenotypes lies in whether mutation-induced perturbation shocks are more likely to be distantly propagated or locally absorbed, as determined by the scaling properties of VSD-induced hydropathic fields (Section 3.2 and SI Fig. S23).

Just as adding a grain of sand to a steep slope can trigger an avalanche, mutations can exploit allosteric shortcuts [82] to augment NaVCh degrees of freedom (DoF), exerting a substantial destabilizing effect on the functional architecture. We conceive the NaVCh functional architecture as a hierarchically organized mechanical system centered around a principal pore axis, where hinges [83], [84], [85], [86] are arranged in nested levels – smaller sub-hinges exist within or regulate parts of larger hinges – thereby enabling multi-scale coordinated motion. Mechanistically, this implies a hierarchy among residues, with some more likely than others to significantly alter channel conductivity and inertia upon mutation-induced perturbation, analogous to how a force applied farther from a hinge generates a larger rotational effect about the axis, which can, in turn, induce large ion/water fluxes along the axis. An in-depth investigation of this idea was undertaken using the analytical procedures detailed in Section 2.1.4. The explanatory strength of this approach is illustrated in Fig. 7 and validated by a simple two-stage scheme reminiscent of meta-learning [87], in which a global model learns across the pore from prior, pore-point-specific (local) learning processes. Our algorithm achieved state-of-the-art AUC scores (relative to previous efforts [35], [65], [88]), demonstrating that a standard support vector machine classifier suffices to learn the biomechanical constraints that differentiate pain-disease-associated mutation hotspots from benign and/or neutral sites. However, it yields only mediocre results when applied to the entire dataset of pathogenic versus non-pathogenic *SCN9A* gene mutations. We argue that this is primarily due to low-resolution artifacts, which affect feature observability, and only secondarily due to limitations of the features themselves.

In summary, relaxing the inertia constraints that lock the transmembrane helical segments around the CC in a screwed-in state is a key perturbation mode through which GoF electrophysiological signatures can arise in the context of human pain disease. An analogous mechanism applies to LoF electrophysiological signatures, but in a more spatially extended form: enhancing rotational freedom at sites along the pore other than the CC can diminish open-state probability. It is therefore reasonable to regard desynchronization as the general mechanistic principle for shifting from a GoF-associated electrophysiological phenotype regime toward a LoF one. Weak desynchronization of transmembrane helical rotations augments channel activation dynamics by increasing the number of accessible PD/VSD coupling configurations, ultimately raising the probability of the open state. Strong desynchronization, however, has the opposite effect: different parts of the transmembrane helical segments begin to behave as asynchronous rotors, effectively decoupling the PD from the VSD and thereby diminishing open-state probability. Targeting NaV1.7’s CC with drug molecules that restore inertial dynamics could therefore serve as an experimental validation strategy. Dismantling orientation and amplitude constraints of HDFs along the extracellular funnel toward the SF constitutes an equally important perturbation mode. Our data suggest that whether a mutation results in gain-of-function (GoF) or loss-of-function (LoF) may depend on the orientation of dehydration forces exerted upon incoming ions (Fig. 7(c) and (d)), a hypothesis that can be tested through molecular dynamics simulations. How these predictions will generalize to larger datasets of GoF versus LoF mutations remains, however, to be determined.

Our findings suggest that evolution has fine-tuned NaVCh metastable dynamics by biasing mutation occurrence toward the outer vicinity of the PD/VSD interface (specifically, on the PD outer boundary described by Eq. (8) and illustrated in Fig. 7). Electrophysiologists can use this principle to interpret and design experiments, while pharmacologists can exploit it to modify NaVCh metastable dynamics in a desired manner. In practice, this means that structurally corresponding sites on the ‘safe-zone’ spherical surface $\partial B_{*}$, located on the extracellular (ES) versus intracellular (IS) side, are nonequivalent and are expected to support distinct perturbation responses. This offers a concrete experimental strategy: electrophysiological assays can systematically target matched sets of residues on $\partial B_{*}$ (e.g., ES-facing versus IS-facing or axially symmetric positions) and compare their effects on activation, inactivation, and use-dependence. Such ‘axial symmetry scans’ effectively mimic the way evolution samples this interface and, under our framework, are predicted to differentially modulate PD/VSD coupling rather than yield uniform outcomes. The most interesting case arises around the AG, where the ‘safe zone’ coincides with the PD/VSD interface. This overlap identifies a spherical shell in which mutations can modulate PD/VSD coupling while remaining preferentially sampled – and apparently tolerated – by evolution, making it an especially attractive region for targeted electrophysiological and pharmacological interventions.

Several limitations apply to our work. First, the structural dataset considered here does not account for the full configuration repertoire that a NaVCh molecule can adopt. Most NaVChs of eukaryotic origin represent snapshots of an inactivated state. Second, we acknowledge that the high-resolution (<3 Å) NaV1.7 structures with PDB codes 8s9b [89], 8xmm [90], and 8thh [91] were not available at the time of structural dataset assembly and are therefore not included in the present analysis. Third, misclassification of VSD hotspots remains an inherent challenge, which could potentially be mitigated by either partitioning the flow into four subflows, each sampling a single VSD, or by initiating RG flows along axes that traverse each VSD individually. As long as Eq. (2) is satisfied, the latter option introduces an axis translation, shifting the perspective from the central pore axis to peripheral VSD-centered axes that simulate the direction along which gating charges move. Lastly, our work does not address dynamic NaVCh aspects, which, in turn, further limits our understanding of hydropathic interaction network intensification characteristics in the VSDs.

In conclusion, the RG provides a coarse-grained yet explanatory framework that efficiently describes NaVCh functional constraints. Leveraging this, machine learning approaches balance computational efficiency with interpretability, enabling clinically relevant insights into pain disorders and advancing understanding of the physicochemical principles shaping NaVCh function. Because our procedures are completely general, they could provide a foundation for analyzing various gene-protein relationships in a pathophysiological context, particularly for large globular or pore-forming systems that are hierarchically organized around a principal point set.

## CRediT authorship contribution statement

**Markos N. Xenakis:** Writing – review & editing, Writing – original draft, Visualization, Validation, Software, Methodology, Investigation, Formal analysis, Data curation, Conceptualization. **Angelika Lampert:** Writing – review & editing, Writing – original draft, Validation, Supervision, Resources, Project administration, Methodology, Investigation, Funding acquisition, Data curation, Conceptualization.

## Funding

This work was funded by the Deutsche Forschungsgemeinschaft (DFG, German Research Foundation) through the following grants awarded to A.L.: 363055819/GRK2415 “Mechanobiology of 3D epithelial tissues (ME3T)”, 368482240/GRK2416 “MultiSenses-MultiScales”, and LA2740/6-1.

## Code availability

The code used throughout is available at: https://github.com/mnxenakis/NaVCh_Scaling

## Declaration of competing interest

The authors declare that they have no known competing financial interests or personal relationships that could have appeared to influence the work reported in this paper.

## Data Availability

The data generated from analyzing 71 prokaryotic NaVCh structures are available at: https://doi.org/10.5281/zenodo.14617204 The data generated from analyzing 50 eukaryotic NaVCh structures and machine learning experiments in the context of human pain disease are available at: https://doi.org/10.5281/zenodo.14628099
